# Hardware Acceleration of Stochastic Neural Networks Enabled by Bit‐Cell Level Co‐Design of Magnetic Tunnel Junctions

**DOI:** 10.1002/advs.78111

**Published:** 2026-09-30

**Authors:** Qiuyuan Wang, Dooyong Koh, Brooke McGoldrick, Tudor Mocioi, Yabin Fan, Marc Baldo, Luqiao Liu

**Affiliations:** ^1^ Department of Electrical Engineering and Computer Science Massachusetts Institute of Technology Cambridge Massachusetts USA; ^2^ Western Digital Corporation San Jose California USA

**Keywords:** magnetic tunnel junctions, probabilistic computing, processing‐in‐memory, spintronics, stochastic neural networks

## Abstract

Processing‐in‐memory architectures mitigate data shuttling bottlenecks in deterministic AI workloads, but still depend on costly external entropy sources for probabilistic models. Here, we present a magnetic‐tunnel‐junction‐based processing‐in‐memory architecture that unifies data storage and massively parallel probabilistic computation for stochastic and deterministic neural networks. By introducing bit‐paired stochastic and stable magnetic tunnel junctions, we realize a multi‐precision stochastic memory cell that transduces stored binary weights into variance‐tunable stochastic bitstreams in situ. We evaluate the architecture's versatility across three progressive regimes of stochastic neural computation. First, by using a hardware‐in‐the‐loop prototype interfacing fabricated stochastic spin‐orbit torque magnetic tunnel junctions with a field‐programmable gate array chip, we validate stochastic matrix‐vector multiplications and achieve near‐floating‐point accuracy without specialized retraining. Second, for networks with stochastic neurons, we demonstrate an Ising machine that utilizes adaptive‐precision sampling and dynamic graph routing to reduce time‐to‐solution. Third, we propose an in‐memory Bayesian computer with independent control of weight means and uncertainties, and apply it to a localization problem under unreliable inputs. By keeping intermediate signals in the stochastic domain, the architecture reduces peripheral overhead associated with random‐number generation and data conversion. This device‐circuit co‐design provides a shared hardware platform for stochastic neural inference, combinatorial optimization, and uncertainty‐aware computing.

## Graphical abstract

An MTJ‐based probabilistic processing‐in‐memory architecture unites nonvolatile storage, local entropy generation, and stochastic computation within each memory cell. Stored multi‐bit weights are converted directly into programmable stochastic bitstreams, enabling neural‐network inference, adaptive Ising optimization, and uncertainty‐aware Bayesian localization with the same device‐circuit primitives while reducing costly data conversion, random‐number generation, and off‐array communication.

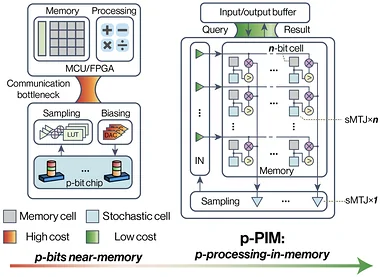

## 1 Introduction

The rapid evolution of data‐centric workloads, from large language models to real‐time sensor fusion, exposes the fundamental limitations of the von Neumann architecture [1–3]. While processing‐in‐memory (PIM) crossbars have effectively mitigated the deterministic “memory wall” by co‐locating matrix‐vector multiplication (MVM) with data storage [4, 5, 6], a subtler yet equally critical “entropy wall” restricts the execution of stochastic algorithms [7, 8, 9, 10, 11, 12]. Modern AI is fundamentally rooted in probabilistic models, relying on statistical algorithms and stochastic sources for generative sampling, combinatorial optimization, and uncertainty‐aware decision‐making [13, 14, 15, 16, 17, 18, 19]. Conventional silicon, however, is deterministic by design. Consequently, simulating probabilistic distributions for these models requires expensive pseudo‐random number generators (PRNGs) and massive data shuttling, which overwhelmingly dominate system energy and latency [20, 21, 22]. Recently, magnet devices have provided encouraging solutions to both the memory and entropy wall problems. Within the scope of PIM, magnetoresistive random‐access memory (MRAM) offers a compelling platform for its high density, endurance, and stability [23, 24, 25, 26, 27, 28, 29, 30, 31, 32, 33, 34, 35, 36]. In parallel, stochastic magnetic tunnel junctions (MTJs) have emerged as ultra‐compact, true random entropy sources, leveraging thermal instability within the standard MRAM material stack [37, 38, 39, 40, 41, 42, 43, 44, 45, 46, 47, 48]. However, current implementations isolate these stochastic primitives from the primary memory fabric (Figure 1a). This separation necessitates continuous data conversion and routing between memory and entropy sources, leaving the entropy wall unresolved.

**FIGURE 1:**
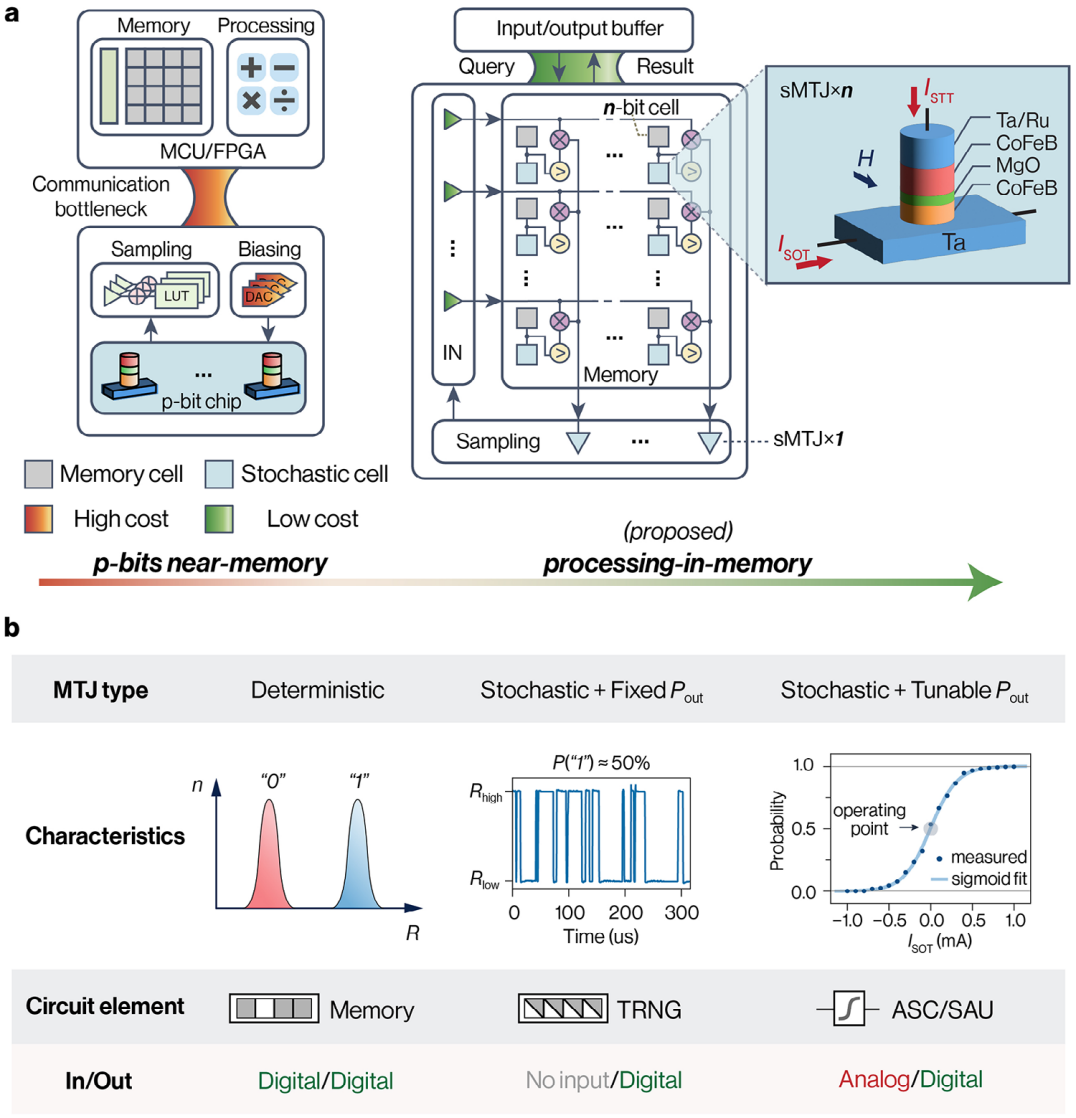
MTJ‐based probabilistic PIM architecture. (a) From near‐memory to in‐memory probabilistic computation (sMTJ: stochastic MTJ). Inset: Fabricated three‐terminal SOT‐MTJ device structure and used material stack. (b) Three CMOS‐compatible MTJ‐based circuit elements as building blocks for bit‐cell level co‐design. Thermally stable MTJs (deterministic) are used as binary memory cells. Thermally unstable MTJs (stochastic) are used as true random number generators (TRNG), analog‐to‐stochastic converters (ASC) or stochastic activation units (SAU) depending on whether the output probability is tuned by an analog voltage. Insets of stochastic MTJ columns are measured from a fabricated three‐terminal SOT‐MTJ device under 35 Oe in‐plane magnetic field.

In this work, we bridge this divide by proposing a versatile MTJ‐based probabilistic PIM (p‐PIM) architecture that unifies deterministic data storage, probabilistic sample generation, and stochastic computing. Moving beyond the usual approach of connecting discrete, standalone physical entropy sources with dedicated peripherals and computing fabrics, we perform a device‐circuit co‐design to achieve an ultra‐high‐density, multi‐precision stochastic memory cell (MP‐SMC) where stochastic MTJs can be integrated directly with deterministic MRAM cells, turning each MRAM storage cell into a multi‐functional probabilistic computing core, while only adding a minimum number of transistors. This cell‐level co‐design allows multi‐bit binary weights stored in MRAM to be transduced into single‐bit‐width, variance‐tunable stochastic bitstreams in situ, as well as performing various stochastic computing operations as needed for different computing tasks. It increases the local density of entropy generation and stochastic computation relative to architectures that separate memory from their entropy sources (Section S3). Further, utilizing three‐terminal spin‐orbit torque (SOT) stochastic MTJs as native analog‐to‐stochastic converters (ASCs) eliminates heavy AD/DA conversion overheads during partial‐sum accumulation [49]. We validate this platform's programmability across three progressive application scenarios with increased demand on entropy generation: executing parallel MVMs for neural network inference, solving combinatorial optimization via Ising spin relaxation, and performing in‐memory sequential Bayesian inference where traditional matrix‐optimized PIMs fail, demonstrating the potential of p‐PIM computation as a scalable solution to expand the boundary of traditional hardware accelerators.

## 2 Device‐Circuit Co‐Design of the Multi‐Precision Stochastic Memory Cell

As shown in Figure 1a, at present the entropy wall is bottlenecking the overall computational throughput of emerging computing systems employing probabilistic bits (p‐bits), analogous to Amdahl's law [50]. An ideal scenario is to integrate p‐bits, memory cells, and computing units directly at the bit‐cell level, forming a processing‐in‐memory platform with intrinsic probabilistic processing capabilities, which we demonstrate can be effectively built from the device‐circuit co‐design of stochastic MTJs and conventional MRAM. Our architecture introduces three MTJ‐based circuit elements by employing MTJs that are thermally stable, thermally unstable with fixed switching probability, and thermally unstable with tunable switching probability (Figure 1b). These distinct MTJ behaviors on the same die can be realized using standard spintronic manufacturing processes to selectively tune the thermal stability factor $\Delta =\frac{E_{b}}{k_{B}T}$, where *E_b_* is the energy barrier between parallel (P) and antiparallel (AP) states. Deterministic MTJs (as standardly used in MRAMs) with high thermal barriers (Δ > 40) are used as binary storage elements. Separately, unstable MTJs are commonly engineered to have Δ < 10 by reducing the free‐layer volume or tuning the magnetic anisotropy. Depending on whether one utilizes an additional input voltage to tune the output probability, the thermally unstable MTJ (stochastic MTJ) can be categorized into fixed (usually 50%) and tunable switching probability. The fixed probability design acts as a very compact true random number generator [51, 52, 53, 54] (TRNG), replacing the bulky linear feedback shift register (LFSR) in digital circuits. The high MTJ resistance and the elimination of an analog input driver make it suitable for generating a uniform stochastic bitstream with high throughput and low power consumption. On the other hand, a stochastic MTJ with tunable probability naturally behaves as an analog‐to‐stochastic converter when collecting partial‐sum accumulation current from a crossbar array. The analog accumulation current *I*_acc_ injected into the heavy‐metal layer of the stochastic SOT‐MTJ is converted to the probability of the device being in the AP state as $P_{AP}\approx \frac{1}{1+\exp(-\alpha I_{\mathrm{acc}})}$, where α is the sensitivity constant depending on the thermal stability factor [49]. To experimentally ground the thermally unstable MTJs, we fabricated three‐terminal, easy‐plane stochastic SOT‐MTJs (Figure 1a, inset) and verified their functionality as both fixed‐probability TRNGs and tunable ASCs (Figure 1b, insets). Here, the TRNGs and ASCs share the same three‐terminal SOT‐MTJ structure. In TRNG mode, the two heavy‐metal channel terminals are shorted, corresponding to the *I*_SOT_ =  0 mA and *P_AP_* ≈ *P_P_* ≈ 0.5 operation point, turning the MTJ into an effective two‐terminal stochastic resistor. In ASC mode, current through the heavy‐metal channel tunes the output probability, while the tunnel junction provides the state readout. Then, by combining these three circuit elements, we propose a scalable, MTJ‐based probabilistic PIM architecture.

The core building block of the p‐PIM architecture is the multi‐precision stochastic memory cell (MP‐SMC), which effectively transforms a passive memory array into an array of very compact probabilistic processing cores (Figure 2a). Here, an *n*‐bit integer weight is stored in *n* stable binary MRAM cells. In each clock cycle, the TRNG block, realized by *n* fixed‐probability stochastic MTJs, generates a uniformly distributed *n*‐bit random number. A local comparator (functional block, circuit shown in Figure 2b) then evaluates these values and outputs a single result bit whose probability of being “1” linearly follows the stored data [55] (Figure 2d,e). Compared with existing approaches to realize multi‐bit stochastic weights – which are either bottlenecked by communication bandwidths when deterministic memory is paired with separated PRNG logic, or suffer from uncontrolled variations when device‐intrinsic and population‐based noise are used – the MP‐SMC overcomes these limitations by executing digital‐to‐stochastic conversion directly within the bit‐cell, combining the advantages of robust digital storage and high‐density, programmable stochastic computation.

**FIGURE 2:**
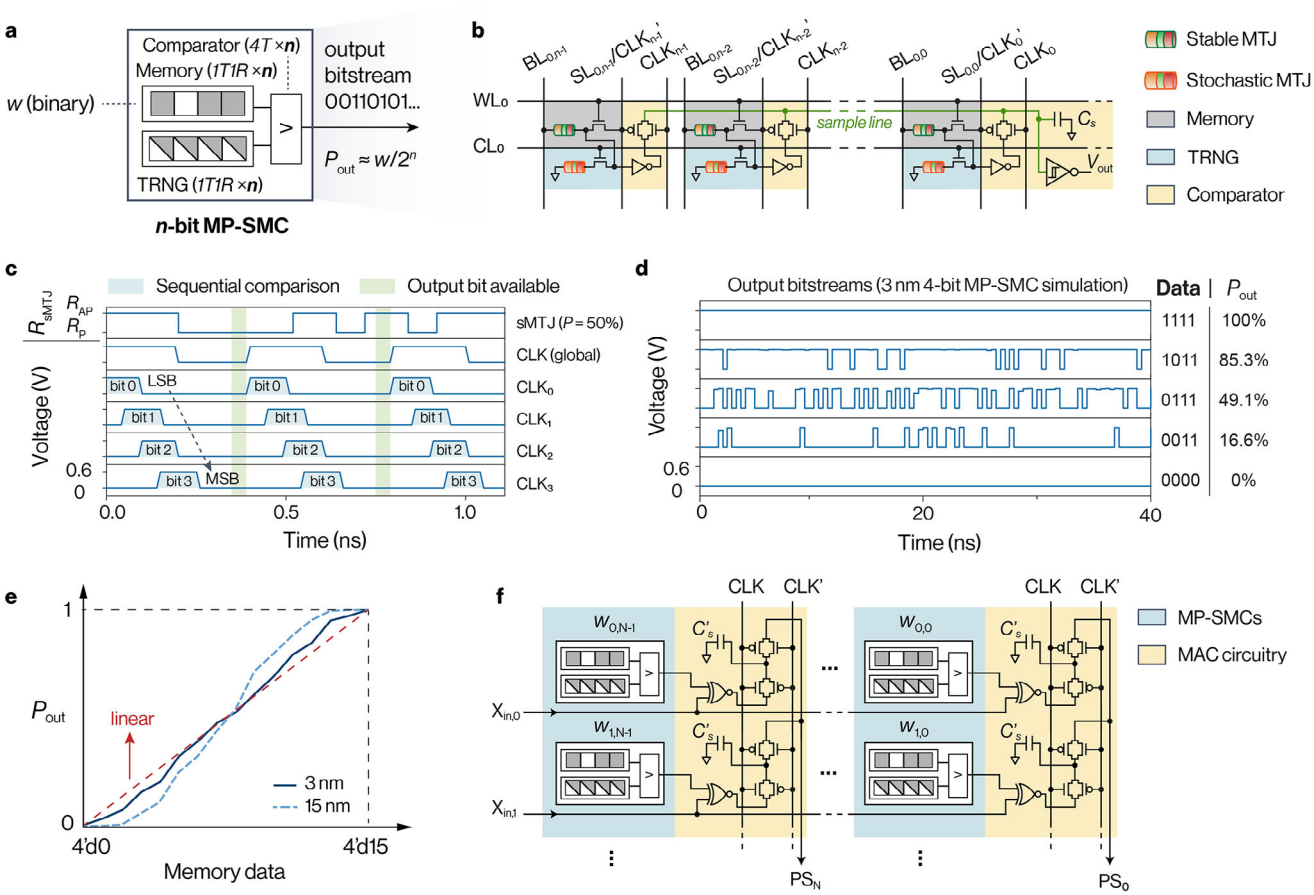
The multi‐precision stochastic memory cell. (a) Functional diagram of an *n*‐bit multi‐precision stochastic memory cell (MP‐SMC) enabling in‐memory binary‐to‐stochastic conversion. (b) CMOS circuit design of the *n*‐bit MP‐SMC (BL: bit line; WL: word line; CL: compute line; SL: sense line; CLK_n_: phase‐shifted clock signals; CLK: global clock signal; C_s_: sampling capacitor). (c) Waveforms of the main global clock, the phase‐shifted clocks, and an example MTJ used in 3 nm node simulation. Output voltage is sampled at the end of each global clock cycle. (d) Simulated output bitstream waveforms of a 4‐bit MP‐SMC with different stored data under 3 nm node. (e) Simulation results showing 4‐bit MP‐SMC output probabilities as a function of memory data under 3 nm and 15 nm technology nodes. (f) In‐memory stochastic matrix‐vector multiplication from an MP‐SMC crossbar array (X_in_: input vector bitstream; PS: output partial sum voltage; MAC: multiply‐accumulate).

The circuit diagram of a 4‐bit MP‐SMC is shown in Figure 2b. When the compute line (CL) is turned off, the MP‐SMC operates identically to a 1T1R MRAM array, guaranteeing reliable data reading and writing. When CL is on, each stochastic MTJ is connected in series with the stable MTJ in the same bit‐cell, acting as a voltage divider. The resulting midpoint voltage $V_{\mathrm{mid}}=\frac{R_{\mathrm{sMTJ}}}{R_{\mathrm{sMTJ}}+R_{\mathrm{MRAM}}}\;V_{BL}$ is then sensed by a 2T inverter and a 2T transmission gate functioning together as a 4T/bit comparator. The comparison logic is executed via the transmission gate (utilizing both NMOS and PMOS to pass full logic and middle‐state voltage swings without threshold drops), driven by phase‐delayed clock lines (CLK_n‐1_ to CLK_0_; waveforms shown in Figure 2c) to open sequentially from the least significant bit (LSB) to the most significant bit (MSB). At each bit step, if the stored bit and sampled TRNG bit differ (here the same AP/P state resistances for the stochastic MTJs and stable MTJs are used, which is physically achievable by engineering the MTJ tunneling layer thickness), the inverter outputs a deterministic high or low voltage to refresh the sampling capacitor with the new comparison result. Conversely, if they are equal, the midpoint voltage *V*_mid_ ≈ 0.5 *V*_BL_ biases the inverter into its transition region, in which neither the NMOS nor the PMOS is fully turned on. The inverter consequently outputs a high‐impedance (high‐Z) middle state, failing to provide enough drive current through the transmission gate, and thus the capacitor holds the previous result. We choose the sampling capacitor value to be large enough to reliably hold the voltage state during the high‐Z condition, yet small enough to rapidly charge or discharge when the inverter outputs a high or low voltage state. The parameter‐variation model and realized distributions are summarized in Tables S1 and S2. The corresponding effects on MP‐SMC transfer characteristics and task‐level performance are presented in Figure S1 and S2, respectively.

In general, an *n*‐bit MP‐SMC contains *n* conventional 1T1R (stable MTJ) storage branches, *n* stochastic 1T1R (stochastic MTJ) entropy branches, *n* 4T comparators (one comparator per bit), a shared sampling capacitor, and a shared 2T output inverter. The total element count is (6*n* + 2) transistors, 2*n* MTJs, and one sampling capacitor per MP‐SMC; for *n* = 4, it corresponds to 26 transistors, 8 MTJs, and one capacitor, giving an effective 6.5T2R bit cell design. Full‐chip bias generation, global routing, input/output buffers, and controls are not included in the area and efficiency analysis.

It is worth noting that while standard MRAM requires massive sense margins to guarantee near‐zero bit error rates (BER), our stochastic architecture inherently tolerates the BER from single‐shot reads because information is encoded in time‐averaged probabilities. Furthermore, instead of operating with a sharp discrimination threshold, the 2T inverter is deliberately designed to work under its low‐gain transition region to establish the high‐Z middle state. SPICE simulations for a 4‐bit MP‐SMC (Methods) validate that this sequential comparison mechanism effectively executes a native Bernoulli draw directly from the stored multi‐bit weight (Figure 2d). We also see that compared with 15 nm technology node, the more advanced 3 nm node, which typically suffers from degraded inverter gain, actually improves the conversion linearity of the MP‐SMC (Figure 2e), a direct benefit of the 2T inverter operating in the transition region.

Using the MP‐SMC as the fundamental building block, we next demonstrate how the p‐PIM architecture accelerates three progressive regimes of stochastic neural computation: from parallel matrix‐vector multiplication for standard neural network inference, to combinatorial optimization using stochastic neurons in an Ising machine, to robust uncertainty‐aware localization using stochastic weights in a native Bayesian accelerator.

## 3 P‐PIM Architecture for Machine Learning Acceleration

Unlike traditional deterministic PIM architectures that are heavily burdened by the area and power overhead of multi‐bit analog‐to‐digital converters (ADCs) and shift‐and‐add logic, the p‐PIM architecture executes parallel matrix‐vector multiplication (MVM) by simply extending each MP‐SMC with a single logic XNOR gate (Figure 2f). In bipolar stochastic computing, real‐valued inputs *x_i_* and weights *w_ij_* (normalized to [− 1,  1]) are represented by time‐domain stochastic bitstreams $X_{i}\left(t\right),W_{ij}\left(t\right)\in \;\left\{-1,+1\right\}$. The exact mathematical multiplication of these values *y_ij_* = *x_i_*  · *w_ij_* is natively executed by the XNOR gate at the cell level: $Y_{ij}\left(t\right)=X_{i}\left(t\right)⊙W_{ij}\left(t\right)$. The spatial accumulation of these bitstreams across a memory column yields the vector multiplication result in the integral stochastic domain $\sum_{i}Y_{ij}\left(t\right)$. Then, this accumulated partial sum, as an analog voltage signal at the bit‐line, drives the stochastic SOT‐MTJ. Since the physical transfer curve $P_{AP}\approx \frac{1}{1+\exp(-\alpha I_{\mathrm{acc}})}$ is identical to the widely used sigmoid activation function, the SOT‐MTJ operates as a stochastic activation unit (SAU) that outputs a new stochastic bitstream for the subsequent layer. Further, by applying a bias voltage across the MTJ pillar, the sensitivity constant α can be tuned [56], enabling the effective activation function to transition continuously between linear, sigmoidal, and binary step behaviors. For applications requiring other non‐linear activation functions, the output stochastic bitstreams can be processed using additional digital peripheral circuitry [57], with the cost of added area, energy, and latency.

By encoding multi‐bit values entirely in the time domain, the p‐PIM architecture inherently bypasses the complex, multi‐cycle shift‐and‐add bit‐slicing logic usually required to realize multi‐bit weight precision in binary memory cells. To validate this architectural advantage, we constructed a hardware‐in‐the‐loop prototype (Figure 3a) where fabricated SOT stochastic MTJs are wire‐bonded to a custom sampling PCB interfacing with an FPGA evaluation board. We program the FPGA so that its circuit implementation strictly corresponds to a 64‐by‐64 MP‐SMC array. The MVM is executed purely in the stochastic domain in the FPGA (Figure 3b), with all random bits for the TRNG blocks collected from a single wire‐bonded physical stochastic MTJ. To overcome the currently low fabrication yield of stochastic MTJs, we use time‐multiplexing to distribute the physically sampled random bits into the corresponding TRNG blocks across the FPGA circuit array. We first evaluated the MVM fidelity using 1000 randomly generated instances with uniform input and weight distributions (Figure 3c). In stochastic computing, multiplication is exact in expectation for uncorrelated input and weight bitstreams. The measured time‐multiplexed sMTJ bitstreams exhibited negligible mean input–weight covariance (Figure S3). Additional simulations quantify the sensitivity of MVM accuracy to array‐wide common‐mode correlation (Figure S6). The standard deviation (σ) of the calculation error that should scale as $\sigma \propto \frac{1}{\sqrt{N_{s}}}$ (*N_s_*: sampling time, in unit of clock cycles) was also explicitly verified on hardware (Figure 3d).

**FIGURE 3:**
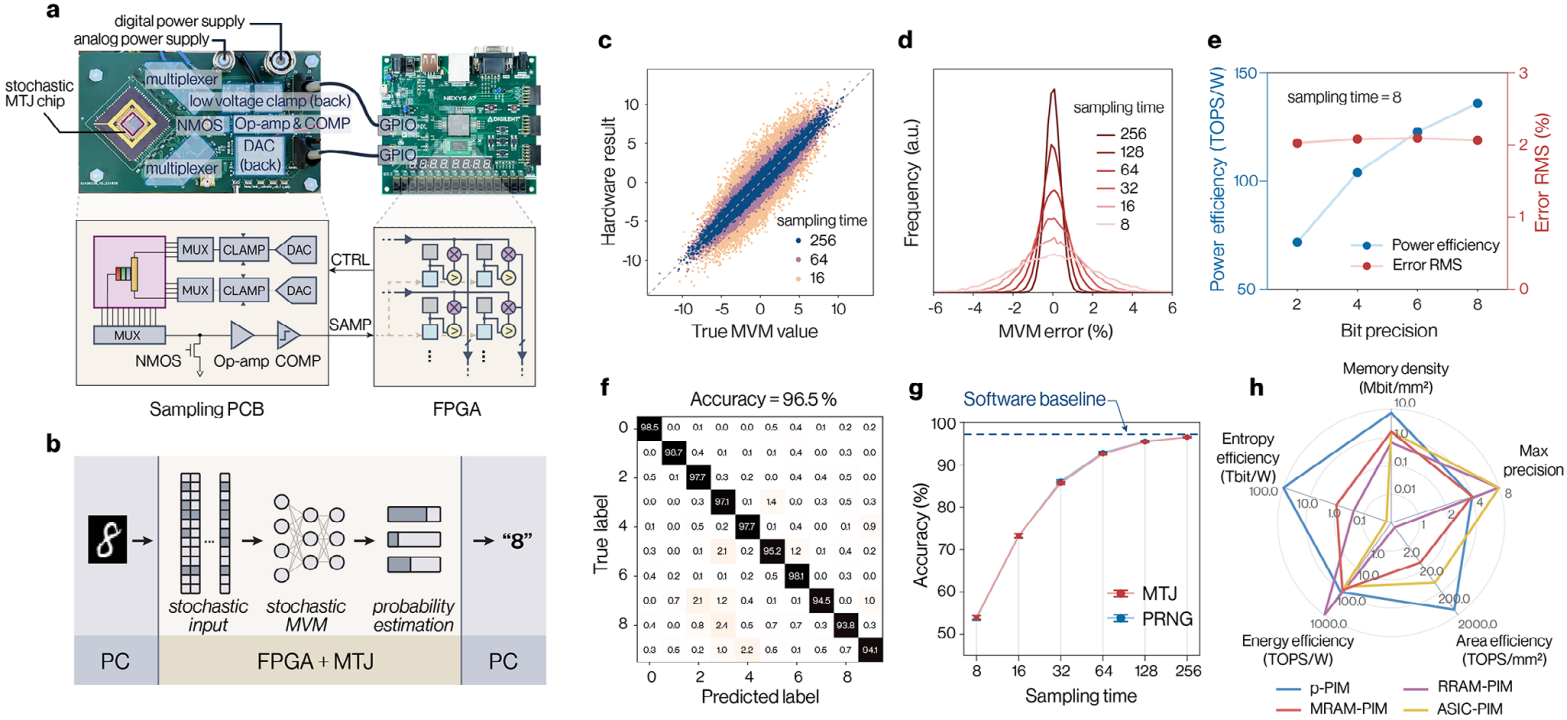
P‐PIM architecture for machine learning acceleration. (a) MTJ‐FPGA system setup. A custom MTJ sampling circuit collects the stochastic bitstream generated by stochastic MTJs. Low‐voltage clamps are used to stabilize the supply voltage and avoid electrostatic discharge. FPGA samples the stochastic bitstream and performs computing tasks queried by a host PC. (b) Machine learning inference experiment process. The PC only performs input data quantization and collects the final inference results. On FPGA, matrix multiplication and activation strictly follow the p‐PIM architecture; the machine learning model is managed by a softcore RISC‐V CPU and C program. (c) Measured MVM results versus true value under different sampling times. Both input and weight elements are randomly generated from uniform distribution ranging from −1 to 1. The matrix height is 64. ASCs are modelled to be biased in the linear region for accurate evaluation of MVM performance. (d) MVM error distribution under different sampling times. (e) The normalized 1b‐1b multiplication power efficiency and relative error root mean square (RMS) as a function of MP‐SMC bit precision. (f) Distribution of MNIST classification results using 4‐bit input and weight precision. A sampling time of 256 is used. (g) Accuracy results as a function of sampling time on 10k MNIST instances over 10 runs. The dashed blue line represents the software baseline on fp32 precision. Stochastic bitstreams from both stochastic MTJ and software pseudo‐random number generator (PRNG) are used and compared. h, Comparison of computing performance between p‐PIM and representative PIM technologies.

Notably, because all arithmetic units—including XNOR gates for multiplication and SAUs for summation and activation—rely on single‐bit logic, their footprint is independent of the underlying MRAM weight precision. As a result, increasing the memory precision favorably improves hardware power and area efficiency, since the size of the arithmetic circuit does not concurrently scale up (Figure 3e). This is also reflected in the normalized computational throughput [4] TOPS_norm_ = TOPS×IN×W×OUT‐ratio (IN and W: the input and weight bit precisions; OUT‐ratio = $\frac{\mathrm{ENOB}}{\log_{2}\left(H\right)+\mathrm{IN}+W}$: the actual output precision divided by the expected output bit‐width; ENOB: effective number of bits derived from output error; H: matrix height).

Next, we deployed a standard 256‐128‐128‐10 multi‐layer perceptron on the MNIST dataset. Because the expected MVM value matches the theoretical output well, the p‐PIM architecture can achieve near‐floating‐point accuracy simply by extending the sampling time, avoiding the need for specialized quantization‐aware training or custom, hardware‐constrained model architecture. The model was trained offline on a PC with fp32 precision using standard deterministic backpropagation, and the weights were directly quantized to 4 bit and sent to the FPGA without any post‐training. The initial image inputs were also converted to stochastic bitstreams by MP‐SMCs within the FPGA. Across 10 runs using all 10 000 MNIST test images, the p‐PIM array achieved 96.52 ± 0.15% accuracy with the measured time‐multiplexed sMTJ as entropy sources and 96.54 ± 0.14% with software generated PRNG bitstreams at *N_s_* =  256 (Figure 3f,g) – both are only 0.7% lower than the ideal software baseline. The effects of bitstream correlation on MNIST accuracy and layer‐wise error propagation are evaluated in Figure S4 and S5, respectively. This approach positions the p‐PIM competitively against existing PIM technologies, delivering a much higher achievable memory density thanks to the low peripheral cost (Figure 3h and Table S3).

## 4 P‐PIM‐Based Ising Machine

While in‐memory MVM acceleration with high memory density effectively addresses the heaviest deterministic workloads in large AI models, we note that utilizing the expected value of long stochastic bitstreams to approximate continuous functions only partially captures the capabilities of the p‐PIM architecture. As modern algorithms increasingly demand the ability to navigate uncertainty, traditional hardware accelerators inevitably collide with the entropy wall—the severe energy and latency penalties incurred by generating, routing, and processing massive amounts of pseudo‐random data. The fundamental advantage of our p‐PIM architecture lies in its ability to dismantle this wall by generating and sampling from tunable probability distributions directly within the memory array. It unifies disparate computing paradigms along a single hardware continuum: progressing from using stochastic bitstreams to represent high‐precision data (stochastic MVMs), to directly exploiting intrinsic per‐bit fluctuations as a thermodynamic driver for state‐space exploration (Ising machines), and ultimately, to natively modulating bitstream variance as a direct measure of epistemic uncertainty (Bayesian inference). To demonstrate this versatility, we now transition to these inherently probabilistic workloads, illustrating how p‐PIM expands the boundaries of traditional computing hardware for stochastic neural networks.

Ising machines are hardware accelerators designed to solve practically important combinatorial optimization problems that can be mapped to an Ising‐type Hamiltonian with *N* interacting spins $H=-\sum_{i,j=1}^{N}J_{ij}s_{i}s_{j}-\sum_{i=1}^{N}h_{i}s_{i},$ where $s_{i}\in \pm 1,J_{ij}$ is the coupling strength matrix, and *h_i_* is the local bias field. Conventionally, in a deterministic computer utilizing the standard Metropolis algorithm [58], updating a spin requires explicitly calculating the energy difference $\Delta E_{i}=2s_{i}\left(\sum_{j=1}^{N}J_{ij}s_{j}+h_{i}\right)$, evaluating the piecewise acceptance probability $P\;\left(s_{i}\to -s_{i}\right)=\min\left(1,e^{-\beta \Delta E_{i}}\right)\;$ (β: inverse temperature), and comparing this high‐precision value against a multi‐bit PRNG. Because this multi‐cycle process demands complex arithmetic operations and incurs massive PRNG overhead, specialized hardware—such as coupled oscillators or bistable circuits [13, 59, 60, 61]—is typically required to realize an efficient Ising machine design. In contrast, the p‐PIM architecture can directly execute this probabilistic Ising spin update without adding niche, application‐specific hardware modifications. In a p‐PIM based Ising machine (Figure 4a), the stochastic SOT‐MTJs behave as Ising spins interconnected by the weight matrix. The MP‐SMC array computes the local field $H_{i}=\sum_{j=1}^{N}J_{ij}s_{j}+h_{i}$ via MVM, and the resulting analog voltage directly drives the MTJ‐based spins. The physical transfer curve of the stochastic MTJ intrinsically acts as the required sigmoid function, causing the spin to flip with the exact Boltzmann probability in a single clock cycle. This eliminates the need to explicitly compute probability amplitudes or rely on digital PRNGs, demonstrating that the p‐PIM architecture provides a unified platform capable of executing both dense MVMs and efficient combinatorial optimization in a single chip.

**FIGURE 4:**
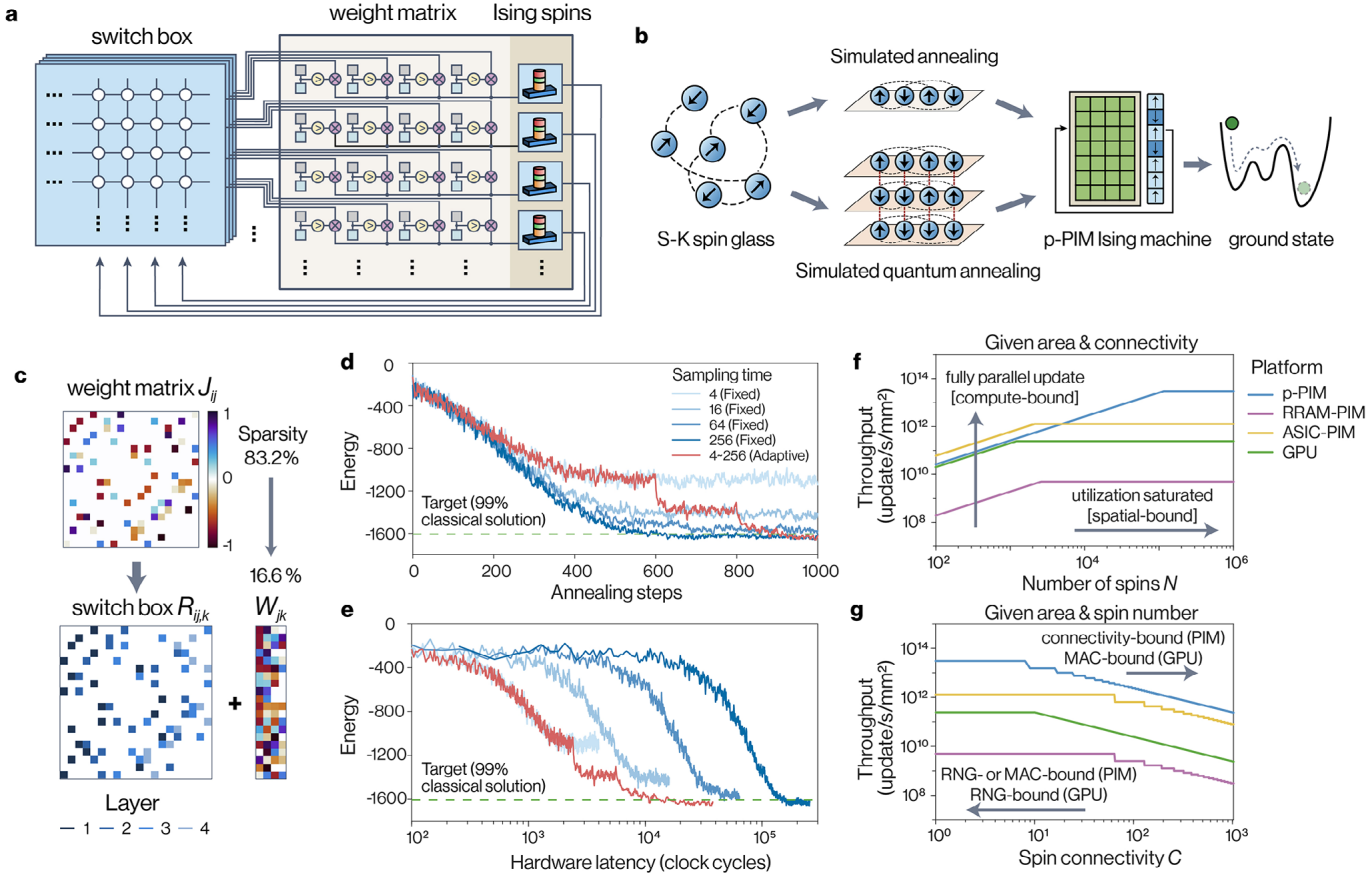
P‐PIM‐based Ising machine. (a) Circuit diagram of a scalable Ising machine implemented on p‐PIM architecture. For an *N*‐spin Ising machine, spin states are sampled and updated in parallel by *N* stochastic SOT‐MTJs interconnected through *C* slices of *N*‐by‐*N* switch boxes, providing all‐to‐all programmability for Ising problems with a maximum connectivity of *C*. The local field is then computed through an *N*‐by‐*C* p‐PIM MVM array. The digital nature of input and output bitstreams allows compact switch box realization such as dense memristor arrays. Multi‐stage switch box topology can further reduce the hardware complexity to O(*N*log(*N*)). (b) Illustration of solving a Sherrington–Kirkpatrick (S–K) spin glass problem. The programmable connection topology allows long‐range coupling and simulated quantum annealing. (c) Example of splitting a sparse weight matrix with a maximum connectivity of 4 into 4 layers of routing matrix for switch boxes and a dense weight matrix of p‐PIM MVM array, reducing sparsity from 83.2% to 16.6% in the MVM core. d,e, Simulated Ising energy evolution as a function of (d) algorithmic annealing steps and (e) hardware latency for a nearest neighbor S–K spin glass problem on a 48‐by‐48 square lattice. The classical solution is obtained from software simulated annealing for 10^4^ steps. The adaptive sampling time method switches sampling time from 4, 16, 64 to 256 on pre‐defined annealing steps. (f) Maximum Ising machine throughput as a function of Ising spin numbers *N* under different hardware platforms, with fixed chip area budget = 1 mm^2^ and spin connectivity *C* = 8. P‐PIM Ising machine is advantageous for large‐scale problems thanks to its high memory density and parallelism. (g) Maximum Ising machine throughput as a function of spin connectivity *C*, with spin numbers *N* large enough to saturate the compute cores. The sampling throughput starts to decrease when memory columns are saturated for PIM architectures (8 for p‐PIM; 64 for RRAM‐ and ASIC‐PIM) or when multiply‐accumulate (MAC) operations take more time than random number generation for GPUs.

A major design constraint for emerging Ising machines, especially in the electronics domain, is the trade‐off between programmability and hardware utilization. The hardware cost of keeping a rigid all‐to‐all connection topology scales as O(*N* ^2^), where *N* is the number of spins. However, real‐world combinatorial optimization problems are typically sparse, with a maximum spin connectivity *C* ≪ *N*. To resolve this, the sparse weight matrix *J_ij_* can be mathematically decomposed into $J_{ij}=\sum_{k=1}^{C}R_{ij,k}W_{kj}\;$, where *W_kj_* is the compressed *C* × *N* dense weight matrix with multi‐bit precision, and *R*_*ij*,*k*_ represents *C* layers of *N* × *N* binary routing matrices that map the active connections. Physically, *R*_*ij*,*k*_ can be realized using programmable switch boxes from compatible non‐volatile memory arrays (Figure 4a). In this way, we map a sparse *N* × *N* problem graph into a dense *C* × *N* p‐PIM execution array (Figure 4c) and greatly increase the utilization rate of the multiply‐accumulate (MAC) resources. Compared to previous works that utilize digital switches or copied nodes to map larger problem graphs onto hardware with lower connectivity [62] or single‐bit weights [63], we extend this concept into the multi‐bit stochastic domain. Because the routed signals are stochastic bitstreams with digital voltage levels rather than fragile analog ones, this programmable routing layer maintains pristine signal integrity even at elevated clock speeds or large circuit noise, establishing a scalable topology for future 3D chip integration.

To validate this approach, we simulated the architecture using a hardware‐parameterized behavioral model to solve a Sherrington‐Kirkpatrick (S‐K) spin glass problem [64] (Figure 4b) on a 48 × 48 square lattice (*C*  =  4) with Gaussian coupling strength $J_{ij}\;∼\;N\left(0,1\right)$ clipped to [− 1, 1]. While traditional solvers usually utilize fixed‐precision arithmetic calculations and apply tunable noise injections to perform simulated annealing, p‐PIM allows for dynamic modulation of the sampling cycles to natively implement the annealing schedule. As shown in Figure 4d, shorter sampling times enable ultrafast, down to single‐clock‐cycle updates with large intrinsic variance, effectively acting as a high thermodynamic temperature to broadly explore the state space and escape local minima; conversely, progressively increasing the sampling time inherently tightens the bitstream variance, mimicking the physical cooling process to achieve high‐precision solution at late stages. This adaptive precision reduces the required hardware clock cycles by more than one order of magnitude compared to fixed‐precision approaches, achieving a well optimized time‐to‐solution (Figure 4e).

The performance for Ising‐type probabilistic state space exploration under various hardware platforms can be summarized by a probabilistic roofline model (Figure 4f,g), which calculates the maximum Ising updates under a strict 1.0 mm^2^ silicon budget with three competing physical constraints: the memory capacity, the compute throughput, and the entropy bandwidth. Traditional architectures hit distinct physical “walls” before saturating their computation potential. Standard GPUs possess high computational capabilities but are severely throttled by shared PRNG bandwidth, creating an entropy wall under large spin number or low spin connectivity. Digital application‐specific integrated circuits (ASICs), burdened by the massive logic area overhead of local LFSRs, hit a capacity wall when spin number increases. Analog PIM arrays offer dense MVM operations and decent storage capacity but are throttled by the low clock frequency due to long RC settling times, leading to low update throughput. By generating robust digital entropy in situ without relying on heavy multi‐bit CMOS logic, the p‐PIM architecture balances computational efficiency with parallel probabilistic sampling, providing a higher system throughput than deterministic computing platforms. A comparison with representative probabilistic‐computing hardware is provided in Table S4.

## 5 P‐PIM‐Based Bayesian Computer

The Ising machine implementation features column‐level parallelism of probabilistic spin flipping, utilizing the stochastic SOT‐MTJs as stochastic neurons. Next, we show that for workloads requiring stochastic weights, the p‐PIM architecture can achieve cell‐level parallelism of probabilistic sampling where each memory cell's expectation value and variance of its target probability distribution can be individually controlled by the stored data bits, turning the memory macro into a Bayesian computer that natively supports Bayesian Neural Networks and sequential probabilistic inference. In contrast, current implementations of Bayesian electronics typically rely on either integrating stochastic physical devices without reliable memory reading or utilizing multiple deterministic memory arrays and post‐processing circuits to produce complex probability distributions [19, 65].

At the core of this capability is the multi‐bit Bayesian cell (Figure 5a), which operates entirely on digitally coded stochastic bitstreams. Comprising two MP‐SMCs, a pass transistor, and a capacitor, the cell enables the simultaneous, independent control of a distribution's expected value (μ) and its variance (σ^2^). The first *µ*‐MP‐SMC directly encodes the state probability into the expected value of its generated bitstream (Figure 5b). Concurrently, the second *σ*‐MP‐SMC drives a compact sample‐and‐hold circuit that modulates the update probability (*P*_update_) of the bitstream, acting as an in‐memory variance controller (Figure 5c). When the *σ*‐MP‐SMC outputs a ‘1’ (opening the gate), the capacitor is refreshed with a new stochastic draw from the first *µ*‐MP‐SMC. Otherwise, the gate is closed, and the capacitor holds its previous state. Functionally, this mechanism scales the effective sampling time of the Bayesian cell—defined as the product of *P*_update_ and the original sampling time—thereby natively adjusting the epistemic uncertainty without altering the mean probability. This dual‐parameter control allows the array to concurrently support continuous learning and Bayesian inference loops (Figure 5d).

**FIGURE 5:**
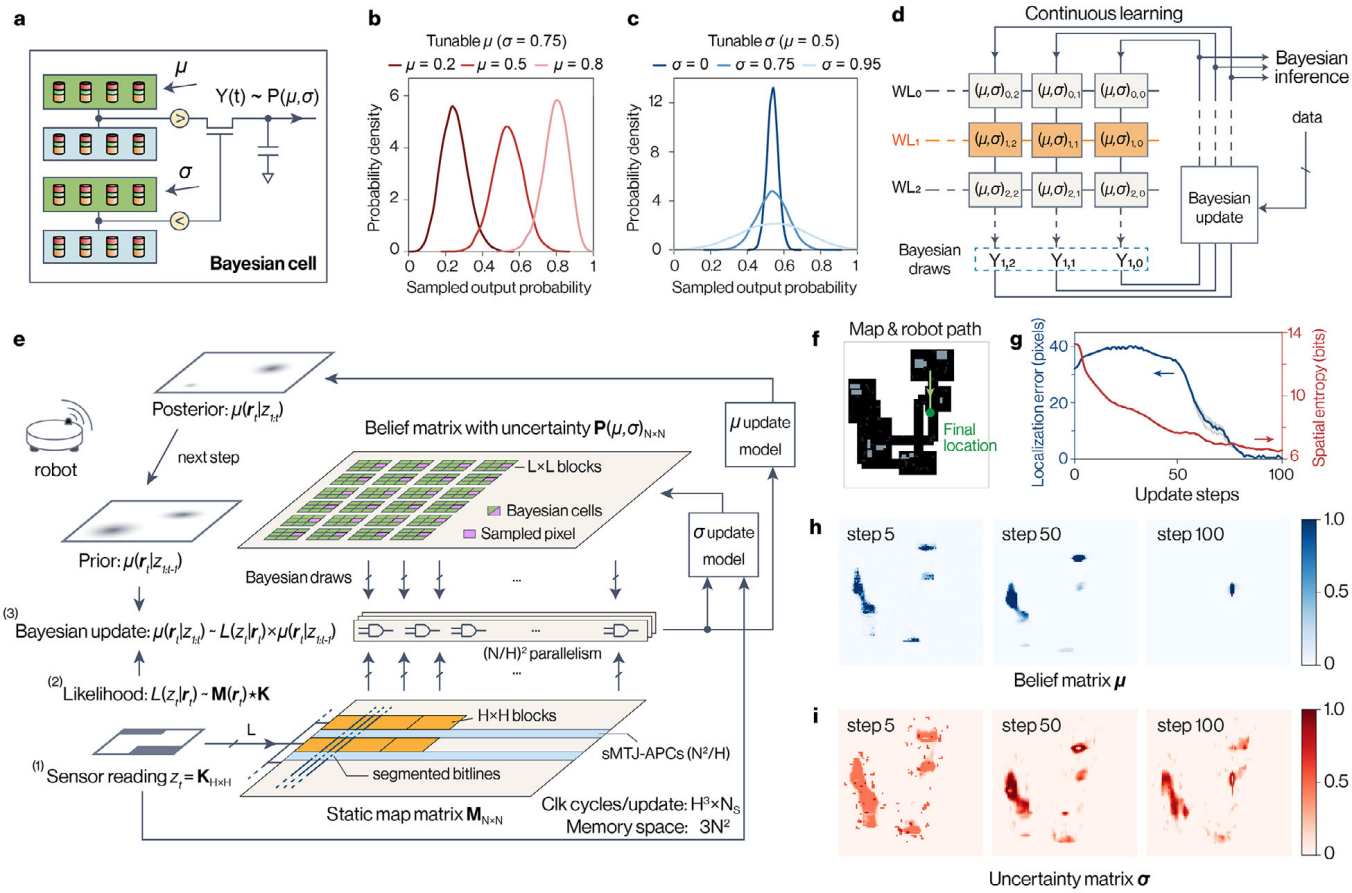
P‐PIM‐based Bayesian computer. (a) Schematic of a 4‐bit Bayesian cell consisting of two 4‐bit MP‐SMCs, one pass transistor, and one capacitor. (b,c) Simulated output probability distribution under different (b) expectation value μ and (c) variance σ with sampling time fixed to 128. (d) Illustration of a Bayesian memory array performing parallel Bayesian draws and updating posterior beliefs for Bayesian inference or continuous learning. (e) Bayesian localization based on the p‐PIM computer. An *N*‐by‐*N* belief matrix with uncertainty *P* is updated recursively as the robot moves and acquires new local sensor readings. The likelihood matrix is calculated by correlating the static map matrix *M* with sensor readings *K*, and parallel pixel‐wise Bayesian update is performed using AND gates. The parallel Bayesian draw and update improves time complexity from O(N^2^) to O(1), without sacrificing space complexity. (f) Map with multiple rooms, obstacles, and detailed features, and the robot path used for hardware‐aware Bayesian localization simulation. (g) Localization error and spatial entropy as a function of update steps over 20 runs (thick solid line: average; faint lines: per run results). (h) Belief matrix under different steps. (i) Uncertainty matrix under different steps.

To validate this in‐memory Bayesian evidence‐fusion framework, we simulated a hardware‐aware robot localization task within a complex, multi‐room environment containing structural ambiguities (Figure 5f). Unlike reinforcement learning or conventional neural network training, which rely on gradient‐based backpropagation, this task performs unsupervised state estimation through recursive Markov localization (Figure 5e). At each time step *t*, the robot moves, acquires a sensor observation *z_t_*, and updates an *N* × *N* belief matrix with uncertainty **P**_*t*_(μ, σ). At each candidate coordinate *r*  = (*x*, *y*) , μ_*t*_(*r*|*z*_1: *t*_) denotes the belief weight and σ_*t*_(*r*|*z*_1: *t*_) represents its uncertainty. The likelihood matrix *L*(*z_t_* |*r_t_*)∝*M*(*r_t_*)⋆*K* is first obtained by correlating the static map matrix *M* with the local *H* × *H* sensor reading *z_t_* ≡ *K*, followed by conversion through the SOT‐MTJ ASCs. Then, parallel logic AND gates produce the unnormalized Bayesian evidence ${\tilde{\mu }}_{t}\;\left(r|z_{1:t}\right)=L\left(z_{t}|r_{t}\right)\mu^{\prime }\left(r|z_{1:t-1}\right)$, where μ′(*r*|*z*_1: *t* − 1_) is the motion‐updated prior. Importantly, to maintain pixel‐level parallelism, each Bayesian cell uses its local evidence bits in a bounded leaky reinforcement/suppression update: supported samples increase the stored belief, rejected samples decrease it, and leakage and saturation constrain the dynamic range. The uncertainty state is updated locally from map reliability and likelihood ambiguity. Normalization is performed only during off‐array readout to compute the reported centroid and spatial entropy, so its cost is not included in the p‐PIM update loop. The complete update rules are given in Section S4.

The localization task results of 20 runs are plotted in Figure 5g. As the robot navigates, the in‐memory architecture sequentially fuses sensor data, resulting in a rapid, simultaneous collapse of both the spatial localization error and the global spatial entropy. The independent modulation of σ provides critical dynamic regularization during inference. Regions of the map with low confidence generate highly variant bitstreams, naturally preventing the Bayesian update mechanism from prematurely converging on erroneous local minima. This mathematical convergence is visually corroborated by the progressive sharpening of the state belief matrix (Figure 5h) and the corresponding localized cooling of the uncertainty matrix (Figure 5i). Ultimately, the p‐PIM architecture successfully tracks hidden states and regulates confidence using a minimal, purely in‐memory footprint.

Because the map correlation, stochastic evidence fusion, and μ/σ‐updates are executed concurrently across ${\left(\frac{N}{H}\right)}^{2}$ processing blocks (Figure 5e), a constant *H* ^3^ *N_s_* clock cycles (*N_s_*: sampling time) latency is achieved for a full‐map update step and is consequently *O*(1) with respect to map size. By contrast, conventional hardware with a fixed number of processing units must evaluate the blocks in successive batches, resulting in an *O*(*N* ^2^) update latency.

## 6 Conclusion

By bit‐cell level co‐design of thermally stable MRAM cells with tunable stochastic MTJs, this work establishes a hardware foundation that directly targets the entropy wall of modern computing. The p‐PIM architecture effectively bridges the gap between high‐density deterministic storage and native probabilistic computation, providing a shared hardware primitive for stochastic neural networks. Instead of shunting data back and forth to external entropy sources or relying on power‐hungry analog‐to‐digital converters, our design confines the entirety of the probabilistic pipeline—from parallel matrix‐vector multiplication to Ising spin updating and pixel‐wise uncertainty regulation—within the memory macro itself. Consequently, the p‐PIM architecture supports multiple workload classes using the same underlying hardware primitives to serve as a versatile, modular computing platform (Figure 6). Utilizing the same MP‐SMC to realize tunable stochastic weights and the SOT‐MTJ to serve as a stochastic activation unit–‐the architecture can be dynamically tailored to execute distinct yet equally important computational tasks, a philosophy similar to recent experimental demonstration of realizing multiple neuromorphic workloads using a single magnetic texture based device [66]. These span from deterministic machine learning via dense MVMs, to combinatorial optimization and physics simulation via Ising spin networks (stochastic neurons), to continuous learning and trustworthy AI via Bayesian inference (stochastic synapses). This multi‐functional modularity establishes a blueprint for fully integrated, probabilistic systems‐on‐chip (SoCs). Within such a framework, interconnected p‐PIM blocks can function as customized computational resources, enabling unified pipelines where high‐throughput sensory feature extraction feeds seamlessly into uncertainty‐aware reasoning and real‐time planning models, all natively in the stochastic domain. Looking forward, the back‐end‐of‐line (BEOL) compatibility of MTJs presents a clear pathway toward 3D monolithic integration, possibly enabling ultra‐high density, massively parallel, energy‐efficient cognitive accelerators capable of resolving complex, real‐world ambiguities directly at the edge.

**FIGURE 6:**
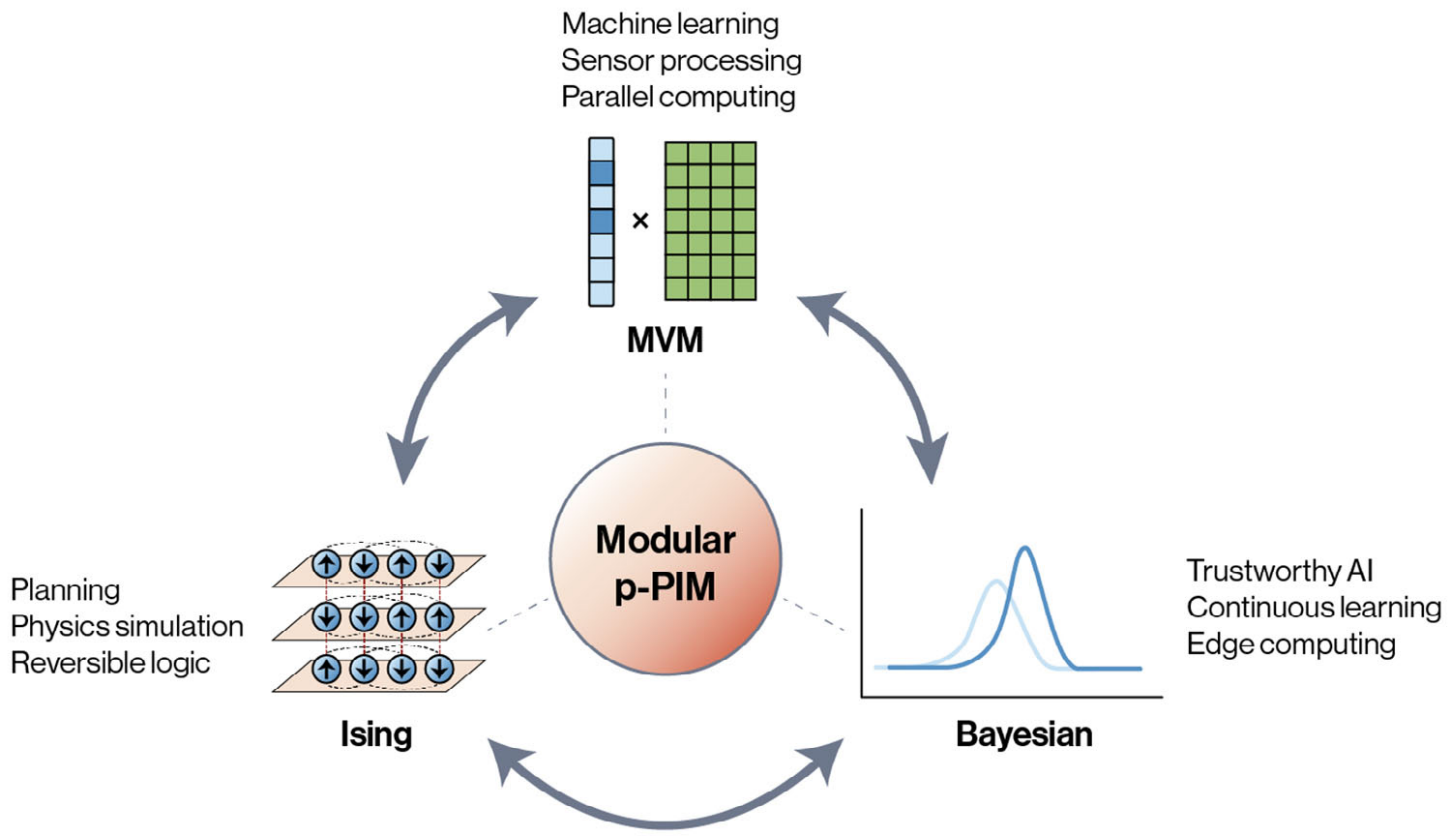
From modular p‐PIM computation to system‐on‐chip. With multiple functionalities demonstrated, including ADC/DAC‐less, high‐density in‐memory MVM, parallel probabilistic Ising update with fully programmable connectivity, and pixel‐wise Bayesian sampling with both probability and uncertainty tunable, the proposed p‐PIM hardware architecture serves as a modular computation platform that can be further tailored to bring passive PIM blocks to fully customizable and interconnected computational resources for building practical multi‐functional system‐on‐chip (SoC).

## 7 Methods

### 7.1 Material Growth and Device Fabrication

The MTJ material stack (from bottom to top; unit in nm, nominal values) Ta(14)/CoFeB(1.3)/MgO(1)/CoFeB(2.6)/Ta(5)/Ru(5) is deposited at room temperature by DC (metallic layers) and RF (MgO layer) magnetron sputtering on a Si/SiO_2_(1 µm) substrate, with a 300°C post‐annealing for 5 mins. Electron‐beam lithography and argon ion milling are used to define the MTJ pillar structure. A SiO_2_ layer deposited by e‐beam evaporation is used for encapsulation. Contact pads are defined by e‐beam lithography, with material layers of Ti (10 nm)/Cu (55 nm)/Au (10 nm) deposited by e‐beam evaporator.

### 7.2 Stochastic MTJ Characterization

After fabrication, standalone MTJ devices are characterized using a probe station. Two Keithley 2400 source meters are used to control the SOT and STT current separately. The voltage across the MTJ pillar is amplified using a low‐noise preamplifier (SR560) and measured by an oscilloscope. An in‐plane magnetic field is applied along the free layer easy axis to bias the device into stochastic switching regime.

### 7.3 Sampling PCB

The sampling PCB is designed to directly feed the stochastic MTJ states to FPGA for architectural level experiments. Multiple MTJs are wire‐bonded onto a chip carrier. Multiplexers (SN74CB3Q3251) around the chip select the MTJ device being sampled. MTJ supply voltages are applied through a digital‐to‐analog converter (DAC121S101) controlled by the FPGA (XC7A100T‐1CSG324C). An operational amplifier (LTC6269) and a comparator (MAX976) are used to amplify and digitize the voltage states from stochastic switching of the MTJ. The digitized signal is sent to FPGA through a GPIO port.

### 7.4 FPGA Programming

The FPGA hardware is programmed using Verilog. Synthesis and implementation are performed using Xilinx Vivado. The FPGA runs under a clock frequency of 50 MHz. An open source 32‐bit RISC‐V CPU (picorv32) is implemented to execute the benchmarking software written in C. A MTJ IO block samples the input waveform from the stochastic MTJ sampling board, converts it to bitstreams and stores the stochastic bits in a cache. The in‐memory‐computing accelerator block contains a 64‐by‐64, 8‐bit precision processing element array for weight storage and matrix‐vector multiplication, as well as a local controller that defines available instructions of the accelerator. On‐chip BRAM is used to store compiled programs and data. A UART block is used for FPGA‐PC communication. A bus connects the RISC‐V CPU with all other blocks through memory‐mapped IO (MMIO). The CPU uses RV32IMC instruction set architecture (ISA). The C programs are compiled using RISC‐V GNU compiler toolchain.

### 7.5 SPICE Simulation

Array‐level circuit simulations are performed in HSPICE using predictive technology models (FreePDK3 and FreePDK15). The stochastic MTJ is modeled using a custom Verilog‐A behavioral model with a 50%–50% P‐AP state probability and an average dwell time of 1 ns (for the 3 nm node) and 2 ns (for the 15 nm node). A device‐circuit co‐design process is implemented to find the optimal transistor sizes and operating points, balancing memory density, computational throughput, and error rate. Device variation and supply voltage fluctuation are considered in SPICE simulation.

## Author Contributions

L.L. and Q.W. conceived the project. Q.W. performed the PCB design, FPGA experiment, and numerical simulations. D.K. fabricated the stochastic MTJ devices with help from B.M. D.K., and Q.W. performed electrical characterization of the fabricated devices. Y.F. provided input on physical realization of the multi‐precision stochastic memory cell. Q.W. and D.K. analyzed the data. Q.W. and L.L. wrote the paper with input from all authors. L.L. and M.A.B. supervised the project.

## Conflicts of Interest

The authors declare no conflicts of interest.

## Supporting information




**Supporting File**: advs78111‐sup‐0001‐SuppMat.docx

## Data Availability

Source data and source codes of this work are available from the corresponding authors upon reasonable request.
