# Supplementary material for: Hardware Acceleration of Stochastic Neural Networks Enabled by Bit‐Cell Level Co‐Design of Magnetic Tunnel Junctions
Source: Adv Sci (Weinh). 2026 Sep 30:e78111. Online ahead of print. doi: 10.1002/advs.78111 (PMC13626169; doi:10.1002/advs.78111)
Supplement: Supplementary file 1 [file ADVS-9999-e78111-s001.docx]

Supporting Information for

**Hardware Acceleration of Stochastic Neural Networks Enabled by Bit-Cell Level Co-design of Magnetic Tunnel Junctions**

Qiuyuan Wang^*,†,1^, Dooyong Koh^†,1^, Brooke McGoldrick^1^, Tudor Mocioi^1^, Yabin Fan^2^, Marc Baldo^1^, Luqiao Liu^1^

^1^Department of Electrical Engineering and Computer Science, Massachusetts Institute of Technology, Cambridge, MA 02139, USA

^2^Western Digital Corporation, San Jose, CA 95119, USA

^†^Equally contributing authors, *email: qiuyuan@mit.edu

**1. Transient simulation and nonideality of MP-SMC**

To evaluate the physical viability and performance of the multi-precision stochastic memory cell (MP-SMC), array-level circuit simulations were performed in HSPICE using FreePDK3 and FreePDK15 transistor models. The 4-bit MP-SMC architecture was simulated under a 0.6 V supply voltage, operating at a 1.25 GHz clock speed for the 15 nm node and 2.5 GHz for the 3 nm node. The stochastic magnetic tunnel junction (sMTJ) models were configured with a fixed 50% output probability for simulation results shown in main text **Figure 2**. The simulated circuit topology directly corresponds to the schematic detailed in Fig. 2b of the main text.

**1.1 Operation principle of MP-SMC**

Standard stochastic computing architectures represent numerical values as expected values of single-bit streams. Traditionally, this requires pseudo-random number generators (PRNGs) coupled with digital comparators^1^—a configuration where substantial area and energy overheads severely restrict parallel sampling. The MP-SMC resolves this bottleneck via a heterogeneous device-circuit co-design that enables *in situ* digital-to-stochastic conversion.

Within each cell, a stochastic sub-circuit (5 transistors, 1 sMTJ) is connected to a conventional MRAM cell (1 transistor, 1 stable MTJ). We introduce a compact 4-transistor sequential comparison circuit—comprising a 2-transistor low-voltage inverter and a 2-transistor transmission gate—to natively transduce the stored *n*-bit binary weight into a single-bit-width stochastic bitstream.

This sequential comparison operates as follows: the resistance of the deterministic MRAM cell ($b_{M}$) is continuously compared against the dynamically fluctuating resistance of the sMTJ ($b_{s}$). A low-voltage inverter evaluates midpoint voltage $V_{\mathrm{mid}}=\frac{R_{\mathrm{sMTJ}}\left( b_{s} \right)}{R_{\mathrm{sMTJ}}\left( b_{s} \right)+R_{\mathrm{MRAM}}\left( b_{M} \right)}V_{BL}$ and maps this input into three distinct states:

- A deterministic logic HIGH when $b_{M}>b_{s}$
- A deterministic logic LOW when $b_{M}<b_{s}$
- A high-impedance (high-Z) state when $b_{M}=b_{s}$

The output bit is maintained by a sampling capacitor, which holds its previous charge state during the high-Z condition. By sequentially sampling the stored bits from the least significant bit (LSB) to the most significant bit (MSB) using phase-shifted clocks, the output probability of the generated bitstream precisely mirrors the multi-bit binary value stored in the MRAM. This approach bypasses both the noisy analog resistive coupling schemes and single-bit limitations characteristic of prior sMTJ-based architectures.

**1.2 MP-SMC non-linearity**

The primary source of non-linearity in the MP-SMC is the leakage current during the high-Z state ($b_{M}=b_{s}$), which slowly discharges the sampling capacitor. Assuming a dominant charging current $I_{c}$ when $b_{M}\neq b_{s}$ and a leakage current $I_{l}$ when $b_{M}=b_{s}$, we can model the voltage decay on the sampling capacitor $C_{s}$. Because the sMTJ switching events follow a Poisson distribution^2^ with an average switching rate $\lambda$ (here we assume that P-to-AP and AP-to-P event have the same switching rate, under $P_{AP}\approx P_{P}=50\%$), the dwell time $\tau$ in the $b_{M}=b_{s}$ state is exponentially distributed. An erroneous bit flip occurs if the capacitor voltage crosses the logic threshold $V_{th}$ before the next evaluation cycle. The threshold crossing time is defined as $t_{c,0\to1}=\frac{C_{s}V_{H}}{I_{l}}$ and $t_{c,1\to0}=\frac{C_{s}\left( V_{DD}-V_{L} \right)}{I_{l}}$, where $V_{H}$ and $V_{L}$ are the threshold voltage for the output comparator (main text Fig. 2b). The induced probability deviation $\Delta P_{k}\left( b_{k} \right)$ under given memory bit $b_{k}$ and bit position $k$ ($k=n-1$ for MSB and $k=0$ for LSB) can be estimated by integrating the probability that the Poisson dwell time exceeds this critical crossing time during the evaluation window $T_{e}$when the transmission gate is open (blue shadow in main text Fig. 2c), as $\Delta P_{k}\left( b_{k} \right)\approx\frac{2^{k}}{2^{n}}\left( -1 \right)^{b_{k}}\int_{t_{c,b_{k}}}^{T_{e}} \lambda e^{-\lambda\tau}d\tau=\left( -1 \right)^{b_{k}}2^{k-n}e^{-\lambda t_{c,b_{k}}}\left[ 1-e^{-\lambda\left( T_{e}-t_{c,b_{k}} \right)} \right]$. Here the factor $2^{k-n}$ accounts for the decreasing weight on the final output bit from the MSB to LSB, and $\left( -1 \right)^{b_{k}}$ accounts for the sign of the erroneous bit flip. As $\Delta P$ will be induced for all *n* evaluation cycles for the *n*-bit MP-SMC, we need to sum up all bits to estimate the final output probability nonlinearity:

$$\begin{aligned} \Delta P\approx\sum_{k=0}^{n-1} \left( -1 \right)^{b_{k}}2^{k-n}e^{-\lambda t_{c,b_{k}}}\left[ 1-e^{-\lambda\left( T_{e}-t_{c,b_{k}} \right)} \right] \#\left( 1 \right) \end{aligned}$$

Advanced technology nodes heavily mitigate this non-linearity. The smoother transfer function of the low-voltage inverter in the 3 nm node broadens the voltage margin for the high-Z state, and lower parasitic capacitance enables higher clock speeds, reducing $T_{e}$ and minimizing vulnerability to leakage.

**1.3 MP-SMC energy breakdown**

The MP-SMC functions as both a high-density memory element and a compute unit, controlled by the compute line (CL) transistor. When CL is pulled low, the PIM circuitry is isolated and grounded, allowing the circuit to operate as a standard MRAM array. Consequently, the p-PIM architecture maintains the same baseline read/write energy efficiencies as traditional MRAM macros. When CL is high and the word line (WL) is low, the sMTJ and evaluation circuits are activated, bring the circuit into the compute mode. In the compute mode, the total energy consumption per cycle $E_{tot}$ is partitioned into static and dynamic components $E_{tot} = E_{stat} + E_{dyn}$. The static energy consumption is dominated by the DC current flowing through the MTJ voltage divider branch during the evaluation cycles $E_{stat}=\sum_{k=0}^{n-1} \int_{0}^{T_{e}} \frac{V_{DD}^{2}}{R_{\mathrm{sMTJ}}\left( t \right)+R_{\mathrm{MRAM}}\left( b_{M} \right)}dt$. The dynamic energy is estimated by the charging and discharging of the effective node capacitances per bit $C_{node}$ and the sampling capacitor $C_{s}$, triggered by sMTJ switching events at an average switching factor $\alpha$ per clock cycle: $E_{dyn}\approx\alpha\left( nC_{node}+C_{s} \right)V_{DD}^{2}$. The energy consumption can be optimized by finding a balanced point across MTJ resistance, sampling speed, supply voltage and output accuracy.

**1.4 Variation-aware simulation of the MP-SMC**

We performed variation-aware transient simulations in HSPICE using the FreePDK15 15-nm transistor model to evaluate the tolerance of the proposed MP-SMC to representative device and circuit variations, including MTJ resistance, TMR, stochastic dwell time, transistor threshold voltage, supply voltage, and passive components. Two Monte Carlo ensembles were evaluated at nominal supply, one at 25 °C and one at 40 °C. Each ensemble contained 256 statistically sampled MP-SMC instances, corresponding to 1,024 stable/stochastic MTJ pairs. Each instance was evaluated at all 16 four-bit input codes. A sample above $0.52\times V_{DD}$ was counted as logic 1, a sample below $0.48\times V_{DD}$ was counted as logic 0, and a sample between these thresholds was marked indeterminate. The mean indeterminate fraction was 0.17% at 25 °C and 0.12% at 40 °C.

**1.4.1 Sampling distributions**

Positive-valued device and circuit parameters were generated using lognormal distributions, while transistor-threshold and supply offsets were generated using normal distributions. Every underlying standard-normal deviate was truncated at $\pm3.5\sigma$. For each Monte Carlo cell realization, the four stable/stochastic MTJ pairs shared a cell-common draw representing a die-level variation. Within each bit position, the stable and stochastic MTJs additionally share a pair-common draw. Separate residual draws were then applied to the two devices. This three-level hierarchy was applied independently to $R_{P}$ and TMR; $R_{AP}$ was then derived according to $R_{AP}=R_{P}(1+\mathrm{TMR}$).

This hierarchical structure produced linear Pearson correlations of 0.953 for $R_{P}$, 0.894 for TMR, and 0.935 for the derived $R_{AP}$ between the stable and stochastic MTJs in each pair. The modeled transistor threshold offset also contained two levels. One global NMOS offset and one global PMOS offset were drawn per Monte Carlo cell and applied to every corresponding transistor block in that cell. Independent local offsets were then added for each inverter, transmission-gate, and readout block. A common supply-scale draw was applied to $V_{DD}$ and $V_{DDL}$ within each cell (here $V_{DDL}$ is the supply voltage to word lines and the low-voltage inverters in compute mode; $V_{DD}$ is the supply voltage to all other components). Sampling-capacitance variation was cell-specific; MTJ capacitance and interconnect resistance were sampled independently for each bit. **Table S1** summarizes the parameter-variation model, and **Table S2** reports the realized parameter distributions for the two 256-cell ensembles.

**Table S1 | Device- and circuit-parameter variations, nominal values, and statistical models.** Cell-common denotes a Monte Carlo draw shared by all four bits within one simulated cell realization but independently redrawn between the 256 realizations. Pair-common denotes a Monte Carlo draw shared by the stable/stochastic MTJ pair within one bit. CV, coefficient of variation.

| **Parameter** | **Nominal value** | **Statistical model** |
| --- | --- | --- |
| Stable/stochastic MTJ $R_{P}$ | 20 kΩ | Lognormal CV: 4% cell-common + 2% pair-common + 1% independent |
| Stable/stochastic MTJ TMR | 100% | Lognormal CV: 5% cell-common + 3% pair-common + 2% independent |
| Stochastic MTJ dwell time | 2.0 ns | Lognormal CV: 2.5% cell-common + 2.5% independent |
| Supply scale | 1.0 | Normal σ = 0.5%; one common scale factor for all four voltage rails in a cell |
| $V_{DD}$ | 0.60 V | Nominal rail multiplied by the common supply-scale |
| $V_{DDL}$ | 0.30 V | Nominal rail multiplied by the common supply-scale |
| NMOS threshold offset | 0 V | Normal σ = 10 mV global + independent σ = 5 mV local per circuit block |
| PMOS threshold offset | 0 V | Normal σ = 10 mV global + independent σ = 5 mV local per circuit block |
| Sampling capacitance | 2 fF | Lognormal CV = 5%, sampled once per cell |
| MTJ capacitance | 0.5 fF | Lognormal CV = 5%, sampled per bit |
| Load capacitance | 0.1 fF | Fixed |

The 40 °C MTJ parameters were generated using the same standardized random draws as at 25 °C and were then scaled using $F_{R}\left( T \right)=1+0.001\left( T-25 \right)$ and $F_{\mathrm{TMR}}\left( T \right)=1-0.0015\left( T-25 \right)$, where $T$ is temperature in °C. Thus, relative to 25 °C, the 40 °C model increases $R_{P}$ by 1.5% and decreases TMR by 2.25%. HSPICE temperature was also set to 40 °C, so the transistor compact model and the explicit MTJ temperature scaling changed simultaneously. The remaining realized circuit-parameter ranges were identical at the two temperatures: $V_{DD}$ ranges 0.5920–0.6085 V; the total NMOS threshold offset covered −31.03 to +36.48 mV and the PMOS offset covered −32.82 to +31.95 mV; the sampling capacitance covered 1.725–2.301 fF; the MTJ capacitance covered 0.420–0.580 fF; sMTJ dwell time including AP/P and P/AP transitions ranged 1.682–2.357 ns.

**Table S2 | Realized parameter distributions.** Each entry is reported as mean [5th percentile, 95th percentile] (minimum, maximum). The resistance and TMR rows contain 1,024 devices of each type at each temperature. The absolute matched-pair mismatch is defined as $\frac{2\left| x_{\mathrm{stable}}-x_{\mathrm{stochastic}} \right|}{x_{\mathrm{stable}}+x_{\mathrm{stochastic}}}$.

| **Quantity** | **25 °C** | **40 °C** |
| --- | --- | --- |
| Stable MTJ $R_{P}$ (kΩ) | 20.05 [18.54, 21.59] (17.72, 23.29) | 20.35 [18.82, 21.91] (17.98, 23.64) |
| Stochastic MTJ $R_{P}$(kΩ) | 20.07 [18.64, 21.62] (17.61, 23.22) | 20.37 [18.92, 21.94] (17.87, 23.57) |
| Stable MTJ TMR (%) | 99.8 [89.8, 109.7] (81.6, 119.1) | 97.6 [87.8, 107.2] (79.7, 116.4) |
| Stochastic MTJ TMR (%) | 99.7 [90.0, 109.4] (80.2, 118.6) | 97.5 [88.0, 107.0] (78.4, 116.0) |
| Stable MTJ $R_{AP}$ (kΩ) | 40.07 [36.39, 43.66] (33.33, 47.22) | 40.21 [36.54, 43.79] (33.48, 47.38) |
| Stochastic MTJ $R_{AP}$ (kΩ) | 40.07 [36.37, 43.60] (34.14, 47.08) | 40.22 [36.52, 43.75] (34.28, 47.23) |
| Pair $R_{P}$ mismatch (%) | 1.122 [0.079, 2.887] (0.002, 5.324) | 1.122 [0.079, 2.887] (0.002, 5.324) |
| Pair TMR mismatch (%) | 2.239 [0.195, 5.566] (0.003, 9.762) | 2.239 [0.195, 5.566] (0.003, 9.762) |
| Pair $R_{AP}$ mismatch (%) | 1.562 [0.131, 4.006] (0.007, 7.020) | 1.553 [0.121, 3.991] (0.002, 6.979) |

**1.4.2 Circuit and task-level performance sensitivity to variations**

The Monte Carlo simulation results for the 4-bit MP-SMC cell are summarized in **Figure S1**. To quantify the application-level consequences of variation-induced MP-SMC probability errors, we repeated the MVM, MNIST, Ising, and Bayesian simulations using the simulated per-code $\Delta P$ distributions. Consistent with our task-level simulations of advanced-node implementation, the nominal MP-SMC was assumed to have a linear binary-to-stochastic transfer curve. The $\Delta P$ contributions arising from device, circuit, and temperature variations were then added to this nominal condition. Full 16-code error distributions were assigned to individual hardware primitives, thereby preserving the correlations among memory bit states within each simulated cell. We performed 20 runs for the MVM, Ising, and Bayesian simulations and 10 runs on all 10,000 test images for MNIST.

The task-level results are summarized in **Figure S2**. For $64\times64$ MVM with sampling time $N_{s}=256$, the full-scale RMSE was 0.723±0.004% for an ideal linear transfer curve, 0.909±0.005% after including device and circuit variations at 25 °C, and 1.589±0.022% after additionally increasing the temperature from 25 °C to 40 °C. MNIST accuracy was 96.5±0.1% for the ideal linear case, 96.1±0.2% with the 25 °C device and circuit $\Delta P$, and 94.7±0.3% for the combined 40 °C condition. In the $48\times48$ Ising benchmark with the same 256 sampling time, the final energy gap relative to the classical simulated annealing result was 0.503±1.234%, 0.559±1.348%, and 1.926±1.147% for the ideal, 25 °C variation-inclusive, and combined 40 °C conditions, respectively. For the Bayesian localization task, the final posterior-mean localization error was 0.20±0.06, 0.19±0.06, and 0.53±0.10 pixels across 20 runs for the corresponding ideal, 25 °C variation-inclusive, and combined 40 °C conditions. Thus, device variation at 25 °C produced little degradation in the Ising and Bayesian localization tasks, suggesting an additional advantage when using p-PIM for probabilistic workloads and by including uncertainty in hardware level.

**Figure S1 |** **Variation- and temperature-dependent MP-SMC transfer characteristics**. **a,** Cell output probability distribution for 256 simulated cells at 25 °C and 40 °C. Each code probability was estimated from 1,024 measured clock cycles. **b,** Cell-paired temperature shift of output probabilities, $\Delta P=P_{40^{\circ}C}-P_{25^{\circ}C}$.

**Figure S2 | Variation-aware task-level simulation results. a,** Full-scale RMSE for $64\times64$ MVMs at $N_{s}=256$. **b,** MNIST classification accuracy results. **c,** Final energy relative to the classical simulated annealing result for the $48\times48$ Ising benchmark at $N_{s}=256$. **d,** Localization error for Bayesian localization.

**1.4.3 Statistical scope and limitations**

The 256-cell ensemble is intended to characterize sensitivity, typical spread, and empirical percentiles. The reported across-cell probability distribution combines parameter variation with a finite 1024 bit length record stochastic sampling due to simulation speed limitation. It should therefore be described as a variation-inclusive finite-trace distribution. Also, the MTJ variation coefficients and correlation hierarchy are engineering assumptions instead of measured wafer statistics. These simulations demonstrate tolerance under the stated model and identify the temperature sensitivity of the in-bit-cell analog-to-stochastic conversion; however, they do not establish manufacturing yield or a guaranteed continuous operating-temperature range.

**2. FPGA-CMOS correspondence**

To bridge the gap between our physical hardware-in-the-loop prototype and the projected full-CMOS integration of the p-PIM architecture, we developed a heterogeneous FPGA+MTJ testing framework. This setup physically validates the stochastic bitstream quality and the algorithmic fidelity of the architecture while emulating the memory and integration mechanics using digital logic.

**2.1 FPGA-MTJ system design**

The hardware-in-the-loop system consists of physical, lab-fabricated stochastic SOT-MTJs wire-bonded to a custom sampling printed circuit board (PCB), which directly interfaces with an FPGA evaluation board (XC7A100T-1CSG324C) operating at a 50 MHz clock frequency. Because the ultra-thin MgO tunneling barrier of the scaled stochastic MTJs yields a low breakdown voltage of approximately 0.2 V, the devices are highly susceptible to electrostatic discharge (ESD). To protect the physical entropy sources, we engineered an active low-voltage clamp (LVCLAMP) circuit that dynamically references an external voltage $V_{\mathrm{ref}}$ and strictly clamps the device voltage to $1.1\times V_{\mathrm{ref}}$. On the sampling board, multiplexers sequence the target MTJs, while onboard DACs supply the required analog voltages to tune the spin-orbit torque (SOT) current flowing through the heavy metal underlayer. An operational amplifier and a high-speed comparator capture the stochastic resistance fluctuations of the active MTJ, amplifying and digitizing these states into 3.3 V logic levels. These true random bitstreams are continuously streamed into the FPGA's General-Purpose Input/Output (GPIO) ports. Within the FPGA, a 32-bit RISC-V softcore CPU (picorv32) orchestrates the computation, communicating with the dedicated p-PIM functional blocks via a memory-mapped I/O (MMIO) bus. The host PC only handles initial data quantization and final result collection via UART, ensuring that all matrix-vector multiplications and probabilistic routing occur natively within the FPGA-MTJ system.

**2.2 MP-SMC comparison**

In the full-CMOS p-PIM architecture, the MP-SMC relies on continuous voltage comparison between deterministic MRAM and fluctuating sMTJ resistances. To accurately emulate this behavior without full monolithic integration, the FPGA isolates the storage and entropy components digitally. The stable MRAM cells are emulated using 8-bit registers, while the physical entropy is supplied by the fabricated SOT-MTJs and buffered in an on-chip cache. The FPGA emulates the binary-to-stochastic conversion using synchronous digital comparators, which possess infinite retention time and zero leakage within a clock cycle. Consequently, the FPGA provides a perfectly linear digital-to-stochastic conversion baseline. While advanced CMOS nodes (e.g., 3 nm) heavily mitigate leakage-induced non-linearity, the FPGA emulation represents an upper bound on algorithmic fidelity unaffected by local capacitor discharge dynamics.

**2.3 ASC comparison**

The analog-to-stochastic converter (ASC) natively leverages the physical characteristics of a tunable three-terminal stochastic SOT-MTJ. As partial sums accumulate as analog voltage on the shared bitline, this voltage drives the stochastic SOT-MTJ and directly modulates its output switching probability to yield a sigmoid-like transfer curve.

In the FPGA emulation, charge sharing is replaced by a digital hierarchical adder tree. To faithfully map this digital integer back to the stochastic domain, the RISC-V CPU queries a hardcoded Look-Up Table (LUT) populated with experimental probability-current transfer curve $P\left( I_{SOT} \right)$ measured from our fabricated device. While physical SOT-MTJs exhibit device-to-device variations, these static variations can be compensated for *in situ* by applying an individual DC bias current to the heavy metal channel or absorbed algorithmically through variation-aware neural network training.

**2.4 Array-yield scaling**

The hardware-in-the-loop system uses one measured sMTJ bitstream interfaced with FPGA storage and stochastic-computing logic. A full $64\times64$ implementation with 4-bit weights will contain 16,384 stochastic MTJs and stable MTJs. Achieving a 50% array yield would therefore require a per-device yield of 99.996% (defect rate of 40 ppm), a reasonable target compared to recent progress in MRAM fabrication process^3^.

**3. P-PIM comparison with conventional PIM and probabilistic-computing hardware**

Here, we compare the proposed p-PIM architecture with representative hardware platforms on the two different workload classes discussed in main text: (1) matrix–vector multiplication (MVM) and neural-network inference, including deterministic analog and digital PIM as well as stochastic and photonic accelerators; (2) probabilistic computing, including p-bit and Ising machines, combinatorial-optimization accelerators, and Bayesian hardware. Quantitative comparison results are summarized in **Table S3** for conventional PIM hardware and in **Table S4** for probabilistic-computing hardware. Here the reported p-PIM density and energy projections include the MP-SMC core devices and local evaluation circuitry represented in the circuit model. Full-chip bias generators, global routing, pads, I/O, and controller overhead are outside this boundary.

**3.1 MVM comparison**

- **Compared with analog-PIM with ADC/DAC:** Analog-PIM requires power- and area-hungry DACs and ADCs. These peripheral blocks dominate system energy/latency and restrict memory density^4^. The p-PIM architecture eliminates ADCs and DACs entirely; utilizing the MP-SMC and ASC, intermediate data remains entirely within the probability-encoded bitstream domain.
- **Compared with digital-PIM:** Processing multi-bit weights in digital-PIM requires complex shift-and-add logic trees, severely limiting parallel compute capacity. By operating in the stochastic domain, p-PIM executes exact mathematical multiplication via a single XNOR logic gate at the cell level. Hardware complexity remains O(1) regardless of weight precision.
- **Compared with analog-PIM with SOT-MTJ based ADC:** Simply replacing a peripheral ADC with an SOT-MTJ fails to resolve input formatting (DACs) and fragile analog accumulation inefficiencies in the core array. The p-PIM architecture is a true device-system co-design that confines the entirety of the probabilistic pipeline within the memory macro.

**3.2 Ising sampling comparison**

- **Compared with digital ASIC and analog PIM:** Digital ASICs are burdened by the massive logic overhead of local LFSRs, hitting a strict capacity wall. Conversely, routing millions of independent noise sources into a dense analog crossbar is physically prohibitive.
- **Compared with specialized continuous-time Ising machines:** Architectures using coupled oscillators exploit physical noise effectively but suffer from severe programmability bottlenecks. Their rigid hardware topologies restrict them to nearest-neighbor graphs, making real-world sparse coupling mapping highly inefficient.
- **The p-PIM advantage:** The p-PIM architecture natively drives the physical transfer curve of the SOT-sMTJ to execute true thermodynamic spin flips in O(1) time. Because spin states and local fields are communicated as robust digital bitstreams, p-PIM circuit can be integrated with programmable non-volatile switch boxes, delivering full all-to-all programmability and adaptive precision scaling without sacrificing memory density.

**3.3 Bayesian electronics comparison**

Current Bayesian hardware implementations rely on integrating stochastic devices without reliable memory reading, or using multiple deterministic arrays with heavy post-processing circuits^5^. The p-PIM architecture introduces a distinct multi-bit Bayesian cell that operates entirely on digitally coded stochastic bitstreams, allowing simultaneous and independent in-memory control of both expected value ($\mu$) and epistemic uncertainty ($\sigma$). This completely bypasses peripheral data movement, natively supporting continuous learning and Bayesian inference loops.

**Table S3 | Comparison of hardware platforms for MVM and neural-network inference.** M, measured hardware; HIL, hardware-in-the-loop; S, circuit/system simulation; P, projection; NR, not reported. Computational throughputs are normalized to 1b as stated in main text.

| **Platform** | **Task** | **Tech.** | **Precision** | **Entropy source** | **Area efficiency** | **Memory density** | **Energy efficiency** | **Accuracy** |
| --- | --- | --- | --- | --- | --- | --- | --- | --- |
| p-PIM (this work) [HIL+S] | MVM; MNIST | 15 nm | 4-bit input and weight | sMTJ | 1214.2 TOPS/mm^2^ | 7.42 Mbit/mm^2^ | 103.9 TOPS/W | MNIST 96.5%;  MVM 0.7231% full-scale RMSE |
| Analog MRAM-PIM^6^ [M] | CNN; CIFAR-10 | 22 nm | 1-bit input, 4-bit weight | Deterministic | 10.90 TOPS/mm^2^ | 1.60 Mbit/mm^2^ | 84.2 TOPS/W | CIFAR-10 90.3% |
| Analog RRAM-PIM^7^ [M] | ResNet-20; CIFAR-10/100 | 22 nm | 8-bit input and weight | Deterministic | 0.32 TOPS/mm^2^ | 0.6667 Mbit/mm^2^ | 998.4 TOPS/W | CIFAR-10 92.01%; CIFAR-100 67.17% |
| Digital CMOS accelerator^8^ [M] | DriveNet; ResNet-50 | 16 nm | 8-bit input and weight | Deterministic | 82.56 TOPS/mm^2^ | 1.613 Mbit/mm^2^ | 61.44 TOPS/W | Full ResNet-50 inference |
| SRAM SCIM^9^ [M] | MVM; MNIST; LeNet-5; CIFAR-10; | 65 nm | 8-bit weight and activation | LFSR | 0.014 TOPS/mm^2^ | NR | 20 TOPS/W | MNIST 99.1%; CIFAR-10 98.4% (top-5) |
| sMTJ stochastic CNN^10^ [S] | LeNet-5-like; MNIST | 22 nm | 4-bit weight | sMTJ | NR | NR | 135.40 nJ/inference | MNIST 97% |
| Chaotic-light photonic computer^11^ [M] | Bayesian LeNet-5 | 4x4 photonic crossbar | Analog | Chaotic light | NR | NR | NR | OOD/ID contrast 25.60x vs 23.24x |

**Table S4 | Comparison of p-PIM with probabilistic-computing hardware.** M, measured hardware; HIL, hardware-in-the-loop; S, circuit/system simulation; P, projection; NR, not reported. Entropy bit rate, spin-update rate, and solution rate are not directly interchangeable.

| **Platform** | **Task** | **Tech.** | **Compute / integration** | **Precision** | **Entropy source** | **Throughput** |
| --- | --- | --- | --- | --- | --- | --- |
| p-PIM (this work) [HIL+S] | SK spin glass; Bayesian localization | sMTJ + FPGA; 15 nm simulated | In-memory | 4-bit input/weight | sMTJ | 2.560 Tbit/s entropy |
| Sparse Ising machine^12^ [M] | Integer factorization; SAT | FPGA | Off-memory; single FPGA | 10-bit weight | LFSR | 1.438×10^11^ flips/s |
| CMOS + sMTJ p-computer^13^ [HIL + P] | Deep Boltzmann-machine | sMTJ + FPGA; 7 nm simulated | Off-memory; sMTJ clocking PRNG | 10-bit weight | sMTJ + digital PRNG | ~1×10^13^ flips/s |
| 80-sMTJ Ising machine^14^ [M] | Travelling-salesman problem | sMTJ + MCU | Off-memory; DAC-connected sMTJs; | Weight NR; 12-bit DAC | sMTJ | 39 solutions/(s ∙W) |
| VCMA-MTJ + p-bit ASIC^15^ [M+P] | Integer factorization | VCMA-MTJ + 130 nm ASIC | Hard-wired weights; off-chip V-MTJs | Static integer weight | V-MTJ | 0.307 kbit/s measured entropy; 10 Mbit/s projected |
| 250-STT-MTJ Ising machine^16^ [M+P] | Integer factorization; MaxCut | STT-MTJ + FPGA | Off-memory; DAC/ADC-connected MTJs | Weight NR; 12-bit DAC | Stochastic STT-MTJ switching | 3.125×10^6^ flips/s measured; ~1×10^15^ flips/s projected |
| Probabilistic-SRAM DCIM annealer^17^ [M] | Travelling-salesman problem | 28 nm | In-memory | 8-bit weight | SRAM read errors | 1.613×10^3^ solutions/s |
| Programmable p-bit computer^18^ [M] | Spin glasses; MaxCut; SAT | Networked FPGA | Off-memory; distributed FPGA coupling | NR | Digital PRNG | >1×10^12^ flips/s |

**4. Hardware-Aware Bayesian Localization Simulation**

The robot-localization simulation was implemented in MATLAB as a hardware-aware behavioral model of the proposed p-PIM architecture. An $N\times N$ grid was used to represent the static map $\boldsymbol{M}$, belief weight $\mu_{t}$, and uncertainty $\sigma_{t}$, with $N=100$. The robot moved through a multi-room environment containing obstacles and grayscale structural features. At each time step, movement along an A*-planned path was enabled with a fixed probability of 0.4. The simulation was performed for 100 update steps, using a fixed sampling time $N_{s}=128$.

| **Algorithm 1: Parallel p-PIM localization update** | |
| --- | --- |
| **Input:** Static map $\boldsymbol{M}$; map-reliability matrix$\boldsymbol{c}_{M}$; previous belief $\mu_{t-1}$; previous uncertainty $\sigma_{t-1}$; robot motion $\Delta\boldsymbol{r}_{t}$; local sensor observation $\boldsymbol{K}_{t}$. | |
| **Output:** Updated belief $\mu_{t}$ and uncertainty $\sigma_{t}$. | |
| **1** | **Motion-prior generation.** When the robot moves by $\Delta\boldsymbol{r}_{t}=\left( \Delta x_{t},\Delta y_{t} \right)$, the belief and confidence fields are spatially shifted and diffused using $D=\left[ \begin{matrix} 0.01 & 0.02 & 0.01 \\ 0.02 & 0.88 & 0.02 \\ 0.01 & 0.02 & 0.01 \end{matrix} \right]$. This produces the motion-updated prior $\mu_{t}^{'}$ and confidence $c_{t}^{'}$, where $c_{t}=1-\sigma_{t}$. If no movement occurs, the previous fields are retained. |
| **2** | **Likelihood generation.** A local $H\times H$ sensor observation $\boldsymbol{K}_{t}$, with $H=17$, is extracted around the true robot position. At each coordinate $\boldsymbol{r}$, correlation between the static map and the sensor observation are accumulated as $I_{t}\left( \boldsymbol{r} \right)=\sum_{u\in\Omega_{H}} \boldsymbol{M}\left( \boldsymbol{r+u} \right)\boldsymbol{K}_{t}(\boldsymbol{u})+\left( 1-\boldsymbol{M}\left( \boldsymbol{r+u} \right) \right)\left( 1-\boldsymbol{K}_{t}\left( \boldsymbol{u} \right) \right)$, where $\Omega_{H}=\left\{ \left( u,v \right):u,v\in\left[ -\frac{H-1}{2},\frac{H-1}{2} \right] \right\}$ is the set of pixel offsets within the sensor window. The normalized correlation current is converted into the likelihood through the SOT-MTJ ASC transfer curve $L_{t}(\boldsymbol{r})=\frac{1}{1+\exp\left[ -\alpha\left( I_{t}\left( \boldsymbol{r} \right)/H^{2}-I_{th} \right) \right]}$, where $\alpha=40$ and $I_{th}=0.85$. The likelihood $L_{t}(\boldsymbol{r})$ is then sampled as stochastic bitstream $S_{t,b}^{L}\left( \boldsymbol{r} \right), b=1,\cdots, N_{s}$ by the ASC. |
| **3** | **Local uncertainty update.** Likelihood values near 0 or 1 indicate an unambiguous match, whereas intermediate likelihoods indicate greater ambiguity. We calculate the observation-derived confidence as $c_{t}^{obs}\left( \boldsymbol{r} \right)= \max\left\{ 0, 1.5\left[ c_{M}\left( \boldsymbol{r} \right)-6L_{t}\left( \boldsymbol{r} \right)\left( 1-L_{t}\left( \boldsymbol{r} \right) \right) \right] \right\}$. Confidence and uncertainty are then updated locally by $c_{t}\left( \boldsymbol{r} \right)=\mathrm{cli}p_{\left[ 0.05,1 \right]}\left( 0.9c_{t}^{'}\left( \boldsymbol{r} \right)+0.1c_{t}^{obs}\left( \boldsymbol{r} \right) \right)$, and $\sigma_{t}(\boldsymbol{r})=1-c_{t}\left( \boldsymbol{r} \right)$. |
| **4** | **Hardware-aware Bayesian draw.** Both $\mu_{t}^{'}\left( \boldsymbol{r} \right)$ and $\sigma_{t}(\boldsymbol{r})$ are quantized to the nearest state of the 4-bit MP-SMC transfer function $Q_{4}$. For each simulated clock cycle, a stochastic belief bit $B_{t,b}\left( \boldsymbol{r} \right)$ and a refresh flag bit $R_{t,b}\left( \boldsymbol{r} \right)$ are generated by performing the Bernoulli draw: $\mathbb{E}\left[ B_{t,b}\left( \boldsymbol{r} \right) \right]=Q_{4}\left[ \mu_{t}^{'}\left( \boldsymbol{r} \right) \right]\mathbb{; E}\left[ R_{t,b}\left( \boldsymbol{r} \right) \right]=Q_{4}\left[ 1-\sigma_{t}\left( \boldsymbol{r} \right) \right]$. The sample-and-hold circuit in the Bayesian cell then gives the prior stochastic bitstream as $S_{t,b}^{\mu}\left( \boldsymbol{r} \right)=R_{t,b}\left( \boldsymbol{r} \right)B_{t,b}\left( \boldsymbol{r} \right)+\left( 1-R_{t,b}\left( \boldsymbol{r} \right) \right) S_{t-1,b}^{\mu}\left( \boldsymbol{r} \right)$. |
| **5** | **Stochastic evidence fusion.** The prior and likelihood bitstreams are fused in parallel using AND gates: $S_{t,b}^{E}\left( \boldsymbol{r} \right)=S_{t,b}^{\mu}\left( \boldsymbol{r} \right)\wedge S_{t,b}^{L}\left( \boldsymbol{r} \right)$, and $\mathbb{E}\left[ S_{t,b}^{E}\left( \boldsymbol{r} \right) \right]=Q_{4}\left[ \mu_{t}^{'}\left( \boldsymbol{r} \right) \right]L_{t}(\boldsymbol{r})$ corresponds to the unnormalized Bayesian evidence. Likelihood-supported and likelihood-rejected prior samples are accumulated as $n_{t}^{+}\left( \boldsymbol{r} \right)=\sum_{b=1}^{N_{s}} S_{t,b}^{E}\left( \boldsymbol{r} \right)$, and $n_{t}^{-}\left( \boldsymbol{r} \right)=\sum_{b=1}^{N_{s}} \bar{S_{t,b}^{E}}\left( \boldsymbol{r} \right)$. |
| **6** | **Bounded local belief update.** Each Bayesian cell updates its belief using only its locally stored state and stochastic bitstreams $\mu_{t}\left( \boldsymbol{r} \right)=\lambda\mathrm{clip}_{\left[ 0,1 \right]}\left[ \mu_{t}^{'}\left( \boldsymbol{r} \right)+\alpha n_{t}^{+}\left( \boldsymbol{r} \right)-\beta n_{t}^{-}\left( \boldsymbol{r} \right) \right]$, where $\lambda=0.995$, $\alpha=1.25/N_{s}$, and $\beta=0.25/N_{s}$. Reinforcement increases the belief at likelihood-supported coordinates, suppression decreases it at rejected coordinates, and leakage and saturation maintain the available cell dynamic range. No array-wide summation, division, or normalized-belief write-back is used in this recurrent update. |

Only for localization result evaluation, the stored belief weights are converted to a probability mass function, $\hat{\mu}_{t}\left( \boldsymbol{r} \right)=\frac{\mu_{t}\left( \boldsymbol{r} \right)}{\sum_{\boldsymbol{r}^{'}} \mu_{t}\left( \boldsymbol{r}^{\boldsymbol{'}} \right)}$. The estimated robot coordinate is calculated from the posterior-weighted centroid,${\hat{\boldsymbol{r}}}_{t}=\sum_{\boldsymbol{r}} \boldsymbol{r}\hat{\mu}_{t}\left( \boldsymbol{r} \right)$, and spatial entropy $\mathcal{H}_{t}=-\sum_{\boldsymbol{r}} \hat{\mu}_{t}\left( \boldsymbol{r} \right)\log_{2} \hat{\mu}_{t}\left( \boldsymbol{r} \right)$. This normalization is not used in the p-PIM belief update loop. The simulation mirrors the functional hardware dataflow: every Bernoulli draw represents an MP-SMC or SOT-MTJ output; belief sampling includes the 4-bit MP-SMC transfer nonlinearity and sample-and-hold temporal correlation; likelihood fusion is performed by bitwise AND gates; belief and uncertainty updates are pixel-local.

**5. Independence of time-multiplexed sMTJ bitstreams and correlation sensitivity**

The hardware-in-the-loop prototype uses one measured sMTJ output sequence to supply stochastic bitstreams for all MP-SMC cells emulated on the FPGA. This prototype is intended to validate device randomness and stochastic computing fidelity given the limited yield of our wire-bonded sMTJ devices.

For the reported experiments, the sMTJ output was sampled at 100 Hz. Across five recordings totaling 2.88 million samples, the lag-1 autocorrelation $\hat{\rho}_{1}=\frac{\sum_{t=1}^{N-1} \left( B_{t}-\bar{B} \right)\left( B_{t+1}-\bar{B} \right)}{\sum_{t=1}^{N} \left( B_{t}-\bar{B} \right)^{2}}$ ranged from -0.00277 to +0.00028 ($B_{t}\in\left\{ 0,1 \right\}$: the $t$-th recorded sMTJ sample). This negligibly small autocorrelation is consistent with an independent, balanced sequence at the 10-ms sampling interval. Temporal correlation can arise when the sampling interval approaches the device dwell time, as measured and discussed elsewhere^19^.

For unipolar stochastic bits, $E\left[ B_{X}B_{W} \right]=p_{X}p_{W}+Cov\left（ B_{X},B_{W} \right）, B\in\left\{ 0,1 \right\}$. For the bipolar stochastic multiplication used in the paper, $Z=\left( 2B_{X}-1 \right)\left( 2B_{W}-1 \right)$, giving $E\left[ Z \right]=xw+4Cov\left（ B_{X},B_{W} \right）$. We therefore evaluated $\mathrm{Cov}\left( B_{X} , B_{W} \right)$ for the 4096 tested input-weight pairs using their corresponding stochastic bitstreams (MP-SMC output given stored data and raw sMTJ bitstreams). As can be seen in **Figure S3**, the mean covariance was $\bar{\mathrm{Cov}\left（ B_{X},B_{W} \right）} =-4.25\times10^{-5}$. This small covariance is consistent with the absence of a resolved systematic MVM bias and the close agreement between the measured RMSE and the theoretical expression.

To understand the computation sensitivity to temporal correlation arising from device nonidealities, high clock frequencies relative to the sMTJ dwell time, or process, voltage, and temperature variation, we next synthesized nonideal stochastic bitstreams with exponential correlation times $\tau=4$, 16, and 64. Their lag-1 autocorrelations were 0.7656, 0.9348, and 0.9830, and the mean matched input-weight covariances were $-7.29\times10^{-6}$, $-3.05\times10^{-4}$, and $-8.58\times10^{-4}$. At $N=256$, MVM RMSE values of 0.7379%, 0.8432%, and 1.5883% full scale and signed biases of +0.0063%, -0.0531%, and -0.1095% full scale, respectively.

**Figure S3 | Input–weight covariance.** Mean covariance $\bar{\mathrm{Cov}\left（ B_{X},B_{W} \right）}$ of using the measured sMTJ bitstreams and the synthesized $\tau$ = 4, 16, and 64 control bitstreams as entropy sources for MP-SMC cells. Colored points represent the mean over 4,096 matched input–weight pairs for each of five recordings or synthetic realizations; diamonds indicate grand means. Error bars are 95% confidence intervals.

Finally, we simulated the 4-bit 256-128-128-10 MNIST network in MATLAB using all 10,000 test images. Ten runs at $N_{s}=256$ gave accuracies of 96.54% for uncorrelated bitstream condition (generated by software PRNG) and 96.52% for the measured time-multiplexed condition (**Figure S4**). Relative to independent finite-stream noise, the measured sMTJ input increased the error in each hidden layer and at the output by only approximately 1.01-fold, indicating that the small covariance did not accumulate appreciably through the network (**Figure S5**). The $\tau=4$, 16, and 64 control bitstreams gave accuracies of 96.50%, 96.17%, and 91.58% and increased the layer errors to approximately 1.02-, 1.17-, and 2.20-fold, respectively. We also note that the accuracy drops for the finite autocorrelation samples are more significant under shorter sampling time, which is a direct result of higher temporal correlation sensitivity for shorter sampling periods.

**Figure S4 | MNIST accuracy sensitivity to bitstream correlation.** Inference accuracy of the 4-bit 256-128-128-10 MLP on 10,000 MNIST test images when using uncorrelated PRNG, measured time-multiplexed sMTJ, and synthesized $\tau$ = 4, 16, and 64 control bitstreams as entropy sources for MP-SMC cells. Error bars are 95% confidence intervals over ten runs.

**Figure S5 | Layer-wise error propagation.** Layer RMSE at sampling time $N_{s}=256$, normalized to the corresponding uncorrelated-PRNG RMSE.

In a future monolithic array, separate sMTJs would eliminate sample reuse but would not exclude common-mode correlation. Possible sources include shared supply impedance, common SOT-bias lines, clock coupling, substrate or interconnect coupling, and so on. We therefore simulated an array-wide common random component while preserving each cell’s Bernoulli marginal. In each cycle, every cell independently used the common random draw with probability $\alpha$ and a local independent draw otherwise. Thus, the probability that two cells used the same common draw was $c=\alpha^{2}$. For this simplified model, the bit covariance of two different MP-SMC cells with output probabilities $p_{i}$ and $p_{j}$ is $\mathrm{Cov}\left( B_{i},B_{j} \right)=\alpha^{2}\left[ \min\left( p_{i},p_{j} \right)-p_{i}p_{j} \right]$ and their correlation $\rho_{ij}=\alpha^{2}\frac{\min\left( p_{i},p_{j} \right)-p_{i}p_{j}}{\sqrt{p_{i}\left( 1-p_{i} \right)p_{j}\left( 1-p_{j} \right)}}$. For 1,000 random $64\times64$ MVMs at $N=256$, the first tested point exceeding a 10% RMSE increase had $c=0.015$, corresponding to $\alpha=12.25\%$ and a mean pairwise Bernoulli correlation of $7.8\times{10}^{-3}$ (**Figure S6**).

**Figure S6 | MVM sensitivity to array-wide common-mode correlation.** MVM RMSE and RMS bias as a function of mean pairwise Bernoulli correlation for 1,000 random $64\times64$ MVMs at $N_{s}=256$. The dashed line marks a 10% increase over the uncorrelated RMSE.

**References**

1.	*Stochastic Computing: Techniques and Applications*. (Springer International Publishing, Cham, 2019).

2.	Funatsu, T., Kanai, S., Ieda, J., Fukami, S. & Ohno, H. Local bifurcation with spin-transfer torque in superparamagnetic tunnel junctions. *Nat Commun* **13**, 4079 (2022).

3.	Ji, Z. *et al.* Achieving high-yield 16 Kb SOT-MRAM array through ion-beam-etched profile control of MTJ cells. *J. Semicond.* **47**, (2026).

4.	Haensch, W. *et al.* Compute in-Memory with Non-Volatile Elements for Neural Networks: A Review from a Co-Design Perspective. *Advanced Materials* **35**, 2204944 (2023).

5.	Querlioz, D. & Vianello, E. Bayesian electronics for trustworthy artificial intelligence. *Nat Rev Electr Eng* **2**, 846–855 (2025).

6.	Deaville, P., Zhang, B. & Verma, N. A Fully Row/Column-Parallel In-Memory Computing Macro in Foundry MRAM With Differential Readout for Noise Rejection. *IEEE Journal of Solid-State Circuits* **59**, 2070–2080 (2024).

7.	Hung, J.-M. *et al.* A four-megabit compute-in-memory macro with eight-bit precision based on CMOS and resistive random-access memory for AI edge devices. *Nat Electron* **4**, 921–930 (2021).

8.	Zimmer, B. *et al.* A 0.32–128 TOPS, Scalable Multi-Chip-Module-Based Deep Neural Network Inference Accelerator With Ground-Referenced Signaling in 16 nm. *IEEE Journal of Solid-State Circuits* **55**, 920–932 (2020).

9.	Yang, J., Li, T., Romaszkan, W., Gupta, P. & Pamarti, S. A 65-nm Digital Stochastic Compute-in-Memory CNN Processor With 8-bit Precision. *IEEE Journal of Solid-State Circuits* **60**, 3749–3761 (2025).

10.	Daniels, M. W., Madhavan, A., Talatchian, P., Mizrahi, A. & Stiles, M. D. Energy-Efficient Stochastic Computing with Superparamagnetic Tunnel Junctions. *Phys. Rev. Applied* **13**, 034016 (2020).

11.	Brückerhoff-Plückelmann, F. *et al.* Probabilistic photonic computing with chaotic light. *Nat Commun* **15**, 10445 (2024).

12.	Aadit, N. A. *et al.* Massively parallel probabilistic computing with sparse Ising machines. *Nat Electron* **5**, 460–468 (2022).

13.	Singh, N. S. *et al.* CMOS plus stochastic nanomagnets enabling heterogeneous computers for probabilistic inference and learning. *Nat Commun* **15**, 2685 (2024).

14.	Si, J. *et al.* Energy-efficient superparamagnetic Ising machine and its application to traveling salesman problems. *Nat Commun* **15**, 3457 (2024).

15.	Duffee, C. *et al.* An integrated-circuit-based probabilistic computer that uses voltage-controlled magnetic tunnel junctions as its entropy source. *Nat Electron* **8**, 784–793 (2025).

16.	Yang, S. *et al.* 250 magnetic tunnel junctions-based probabilistic Ising machine. *Nat Commun* **17**, 5368 (2026).

17.	Kong, Y. *et al.* A compact digital compute-in-memory Ising annealer with probabilistic SRAM bit in 28 nm for travelling salesman problem. *npj Unconv. Comput.* **3**, 15 (2026).

18.	Aadit, N. A. *et al.* Programmable Probabilistic Computer with 1,000,000 p-bits. Preprint at https://doi.org/10.48550/arXiv.2606.25313 (2026).

19.	Koh, D. *et al.* Closed Loop Superparamagnetic Tunnel Junctions for Reliable True Randomness and Generative Artificial Intelligence. *Nano Lett.* **25**, 3799–3806 (2025).
